# Ergosterol peroxide from marine fungus *Phoma sp*. induces ROS-dependent apoptosis and autophagy in human lung adenocarcinoma cells

**DOI:** 10.1038/s41598-018-36411-2

**Published:** 2018-12-18

**Authors:** Han-Ying Wu, Feng-Ling Yang, Lan-Hui Li, Yerra Koteswara Rao, Tz-Chuen Ju, Wei-Ting Wong, Chih-Yu Hsieh, Michael V. Pivkin, Kuo-Feng Hua, Shih-Hsiung Wu

**Affiliations:** 1 Institute of Biological Chemistry, Academia Sinica, Taipei, Taiwan; 2 Chemical Biology and Molecular Biophysics Program, Taiwan International Graduate Program, Academia Sinica, Taipei, Taiwan; 3 Department of Chemistry, National Tsing Hua University, Hsinchu, Taiwan; 4 Department of Laboratory Medicine, Linsen, Chinese Medicine and Kunming Branch, Taipei City Hospital, Taipei, Taiwan; 5 Department of Biotechnology and Animal Science, National Ilan University, Ilan, Taiwan; 6 Department of Animal Science and Biotechnology, Tunghai University, Taichung, Taiwan; 7 Graduate Institute of Life Sciences, National Defense Medical Center, Taipei, Taiwan; 8 G.B. Elyakov Pacific Institute of Bioorganic Chemistry FEB RAS, Vladivostok, Russia; 9 Department of Pathology, Tri-Service General Hospital, National Defense Medical Center, Taipei, Taiwan; 10 Department of Medical Research, China Medical University Hospital, Taichung, Taiwan

## Abstract

As part of our ongoing search for novel therapeutic structures from microorganism, the chemical examination of marine fungus *Phoma sp*. resulted in the isolation of ergosterol, ergosterol peroxide (EP), and 9,11-dehydroergosterol peroxide (DEP). The bioassay results demonstrated that the three isolates reduced the viability of various cancer cells, with EP being highest in human lung cancer cell line A549 cells. EP induced caspase-dependent apoptosis through mitochondrial damage in A549 cells. Additionally, EP-induced ROS generation and apoptosis were attenuated by ROS-generating enzymes inhibitors and antioxidant *N*-acetylcysteine, indicated that ROS played an important role in EP-mediated apoptosis in A549 cells. Furthermore, it was observed that EP induced ROS-dependent autophagy, which attenuated apoptosis in A549 cells. On the other hand, EP reduced the LPS/ATP-induced proliferation and migration of A549 cells through attenuated NLRP3 inflammasome activity. Additionally, EP showed synergistic cytotoxic effect with antitumor drug Sorafenib in A549 cell viability inhibition. Furthermore, Micro-Western Array and Western blot analyses demonstrated that the protein levels of EGFR, HSP27, MEK5, AKT1, mTOR, Smad2, Smad3, TAB1, NF-κB, and HIF1-α decreased, while the levels of p-p38α, p-ERK1/2, p-JNK, fibronectin and p27 increased. Collectively, the results of this study demonstrated that EP might be useful to develop a therapeutic candidate for lung cancer complications.

## Introduction

The incidence of cancer is the most serious health issues of the 21^st^ century, with lung cancer being the leading cause of cancer-related deaths throughout the globe. The main types of lung cancer are non-small cell lung cancer (NSCLC) and small cell lung cancer. The major lung cancer NSCLC includes various types such as squamous cell carcinoma, large cell carcinoma, adenocarcinoma, pleomorphic, carcinoid tumor, and salivary gland carcinoma^1^. The metastasis-related death found to be as high as 90% of all lung cancer mortality^2^. Despite advances in conventional treatment procedures such as surgery, radiation, chemotherapy and targeted therapy, however, the clinical outcomes of the current therapies are still not at satisfactory level. Regardless of lung cancer subtypes, the overall 5-year survival rate of lung cancer is ~15%^3^. Furthermore, the conventional treatment methods including surgery, radiation and chemotherapy are associated with unwanted side effects such as pneumonitis, nausea, decreased blood cell counts, hair loss and mouth sore^3^. Therefore, the identification of novel anticancer agents to minimize the chemotherapy side effects and to overcome the inherent resistance against therapeutic drugs is eagerly needed^4^.

Natural products and derivatives are fascinating molecules for modern drug discovery and development^5^. Microorganisms, including bacteria, fungi and microalgae are rich source for novel structures and contribute greatly in the discovery of new pharmaceuticals^6^. In particular, the fungi provide a great chemical diversity and exhibit interesting pharmacological properties including the inhibition of NSCLC^7^. *Phoma* is a ubiquitous and widespread fungus with species found in soil^8^. It is known that the lipophilic crude extract of *Phoma sp*. shows anti-lung cancer activity^9^, however, little known about the target compounds and their molecular mechanisms against NSCLC.

Cancer cells develop various strategies to limit their death, and unregulated apoptosis is an important hallmark in cancer cells death^10^. The dysregulation of mitochondria function is one of apoptosis incentive. Generation of reactive oxygen species (ROS) conventionally regarded as cytotoxic and apoptosis inducers in cancer cells^10^. Autophagy (self-eating) is a highly conserved catabolic process that facilitates nutrient recycling through lysosomal mediated degradation. The base level autophagy is necessary to maintain normal cellular homeostasis. However, in cancer cells autophagy may play a role as both a tumor suppressor and a cell survival inducer. Recent studies demonstrate that ROS activate the autophagy in various stimulating conditions^11,12^. On the other hand, inflammation is defense mechanism in living organism, however, persistent and chronic inflammation play an important role in cancer development including lung cancer^13^. The elevated NLRP3 inflammasome identifies as a cell migrator and proliferator in lung cancer^14^. This study was undertaken as part of our continuous search for the identification of therapeutic structures against lung cancer. In this study, HPLC-UV-guided fractionation and purification of the ethyl acetate extract from the solid substrate fermentation cultures of *Phoma sp*., led to the isolation of metabolites ergosterol, ergosterol peroxide (EP), and 9,11-dehydroergosterol peroxide (DEP). The isolated metabolites examined for their cytotoxic potential in different cancer types as well as normal cells. The potential cytotoxic agent EP against A549 cells further evaluated for its ability to induce apoptosis, autophagy, ROS generation, and to attenuate the elevated NLRP3 inflammasome in A549 cells.

## Results

### Cytotoxic potential of Phoma sp. isolates

**Figure 1:**
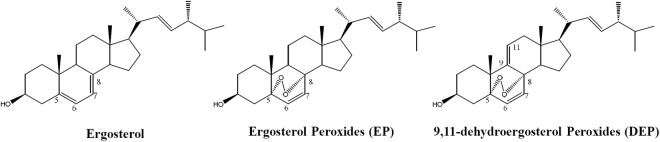
Chemical structure of ergosterol, ergosterol peroxide (EP) and 9,11-dehydroergosterol peroxide (DEP) isolated from *Phoma sp*.

At first, the cytotoxic potential of *Phoma sp*. metabolites (Fig. 1), ergosterol, ergosterol peroxide (EP), and 9,11-dehydroergosterol peroxide (DEP) was evaluated in different type of cancer cell lines A549, J5, HeLa and MCF-7, and normal lung cell line Beas-2b, as well as macrophages RAW 264.7. The cells exposed to each compound individually with increasing concentrations for 24 h, and then the cell viability was determined by MTT assay. Our results demonstrated that the tested compounds showed varied inhibitory potency on viability of different cell types with IC_50_ values ranged from 14 to 222 µM (Table 1). It was noted that EP showed superior inhibitory potential as compared with ergosterol and DEP, being maximal against the lung cancer cell line A549 (IC_50_ = 23 µM), followed by MCF-7, HeLa and J5 cells (Table 1). The compounds ergosterol and DEP responded differentially to different cancer types where the former potently inhibited J5, followed by HeLa, A549, and MCF-7 cells, while the later showed the same against MCF-7, J5, HeLa and A549 cells, respectively. In particular, ergosterol showed more cytotoxic effect in J5 and HeLa cells with an IC_50_ value of 14 and 19 µM, respectively, as compared to the A549 (IC_50_ = 35 µM) and MCF-7 (IC_50_ = 48 µM) (Table 1). DEP exhibited moderate cytotoxicity against tested cancer cells with IC_50_ value ranging from 34 to 49 µM. On the other hand, our results showed that ergosterol and DEP were nontoxic to normal lung cells Beas-2b, as well as macrophages RAW 264.7, while EP showed less sensitive (i.e., IC_50_ values 174 and 222 µM, respectively) (Table 1). These results pointed that the tested compounds ergosterol, EP and DEP were more sensitive to cancer cells, while less sensitive or nontoxic to normal cells. As the aim of this study was to identify potential lung cancer cells inhibitors, subsequently, the compound EP was used to evaluate the underlying molecular mechanism of its superior inhibitory potential against A549 cells growth.

**Table 1:** Cytotoxicity of Ergosterol, EP and DEP against cancer cells.

^a^IC_50_	Ergosterol	EP	DEP
HeLa	19 ± 5.4 μM	31 ± 1.8 μM	37 ± 1.4 μM
A549	35 ± 2.6 μM	23 ± 1.5 μM	49 ± 2.7 μM
J5	14 ± 6.8 μM	33 ± 2.8 μM	36 ± 1.9 μM
MCF-7	48 ± 1.3 μM	29 ± 3.1 μM	34 ± 1.8 μM
Raw264.7	Non-determined	174 ± 27.6 μM	Non-determined
Beas-2b	Non-determined	222 ± 22.6 μM	Non-determined

^a^IC_50_ value expressed as the mean ± SD of three independent experiments.

### EP induced apoptosis and inhibited colony formation in A549 cells

**Figure 2:**
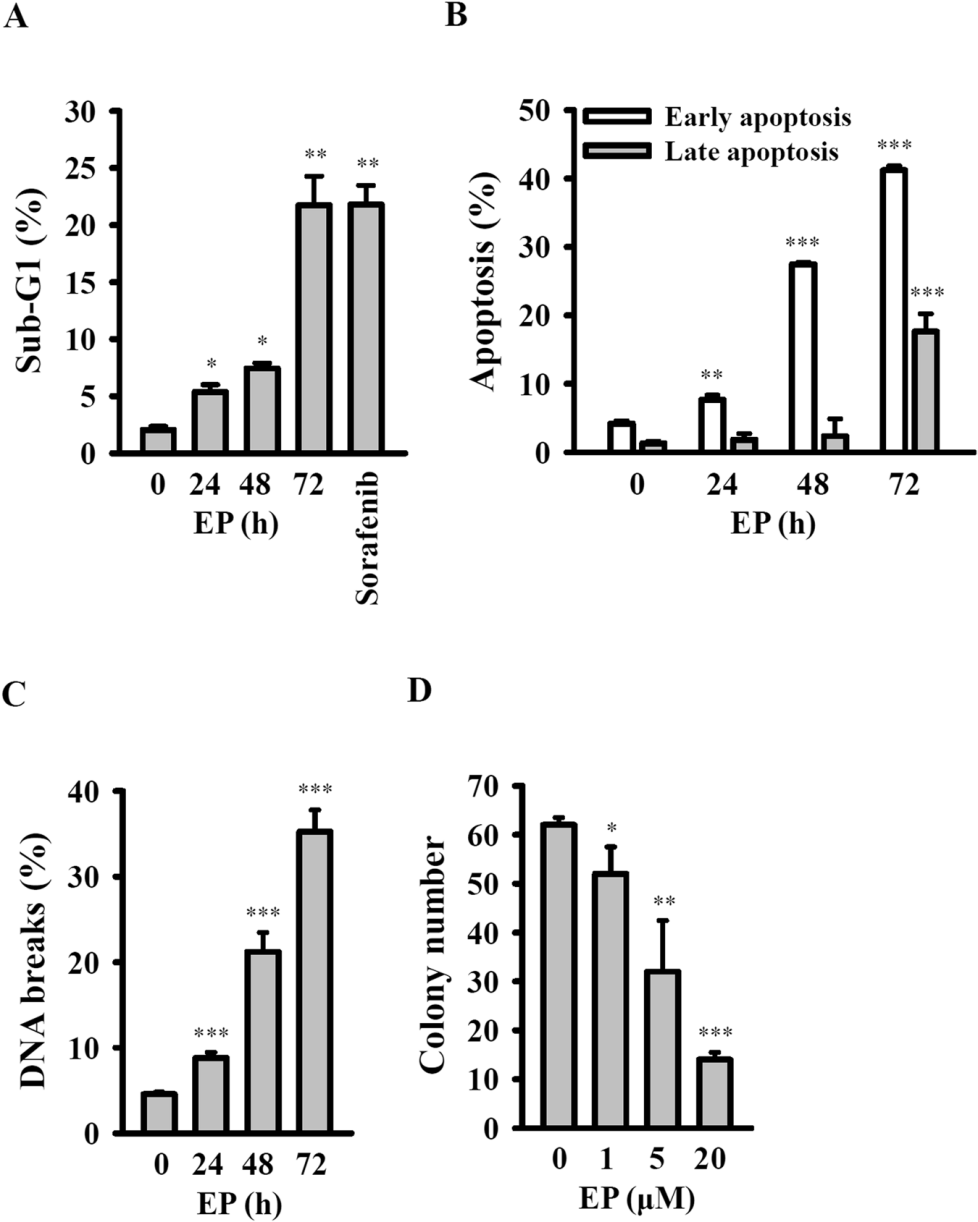
EP induced apoptosis and inhibited colony formation in A549 cells. (**A**) Cells in sub-G1 phase assayed by PI staining. (**B**) Apoptosis assayed by PI and Annexin V double staining. (**C**) DNA breaks assayed by TUNEL assay. (**D**) Colony formation assay. *^,^** and *** indicate significant differences at the levels of *p* < 0.05, *p* < 0.01 and *p* < 0.001, respectively, compared to control cells.

To evaluate whether or not EP induced apoptosis in A549 cells, we analyzed the cell-cycle distribution by propidium iodide (PI) staining. The cell cycle analysis results showed that after treatment with EP or Sorafenib (Raf kinase inhibitor)^15^, the A549 cells in sub-G1 phase enhanced in a time-dependent manner (Fig. 2A and Sup. Fig. 1A). In particular, when A549 cells were treated with 20 µM of EP, the percentage of Sub-G1 phase cells increased from a control value of 2.3 to 5.2, 7.5 and 22.3% for time corresponding to 24, 48 and 72 h, respectively (Fig. 2A). To confirm the effect of EP on A549 cells apoptosis, Annexin V/PI double staining method performed and analyzed by flow cytometry^16^. The results revealed that exposure to 20 µM of EP, the percentage of early-stage A549 apoptotic cells increased from the control value of 3.2 to 8.7, 28.6 and 42.7% after 24, 48 and 72 h, respectively (Fig. 2B and Sup. Fig. 1B). In contrary, it was noted that there was no significant difference in the late-stage A549 apoptotic cells up to 48 h, however, increased the same from the control value of 1.9 to 19.7% after 72 h (Fig. 2B and Sup. Fig. 1B). These results indicated that EP not only induced the early stage of apoptosis at 24 h but also caused the late stage of apoptosis at 72 h. Moreover, the apoptosis inducing effect of EP in A549 cells was investigated by DNA breaks assay. Our results showed that the percentage of cell with DNA breaks enhanced from the control value of 4.6 to 9.4, 22.8 and 36.4% after 24, 48 and 72 h, respectively (Fig. 2C and Sup. Fig. 1C). Additionally, it was also noted that EP dose-dependently inhibited the colony formation ability of A549 cells with an IC_50_ value of ~5 µM. In particular, A549 cells colony formation reduced from the control value of 62.1 to 9.8, 31.4 and 15.3% by 1, 5 and 20 µM of EP, respectively (Fig. 2D and Sup. Fig. 1D). On the other hand, we also examined the effect of EP in different lung cancer cell lines with different metastatic properties. The results from Annexin V/PI double staining method showed that 20 µM of EP induced apoptosis in non-metastatic CL1-1 cells (Sup. Fig. 1E). The sub-G1 phase (Sup. Fig. 1F) and DNA breaks (Sup. Fig. 1G), of CL1-1 and CL1-5 cells were increased by 20 µM of EP, with CL1-5 was more susceptible to EP treatment. By HPLC separation, we also confirmed that EP indeed entered into cytosol of A549 cells (Sup. Fig. 2).

### EP induced mitochondrial damage and caspase-dependent apoptosis

**Figure 3:**
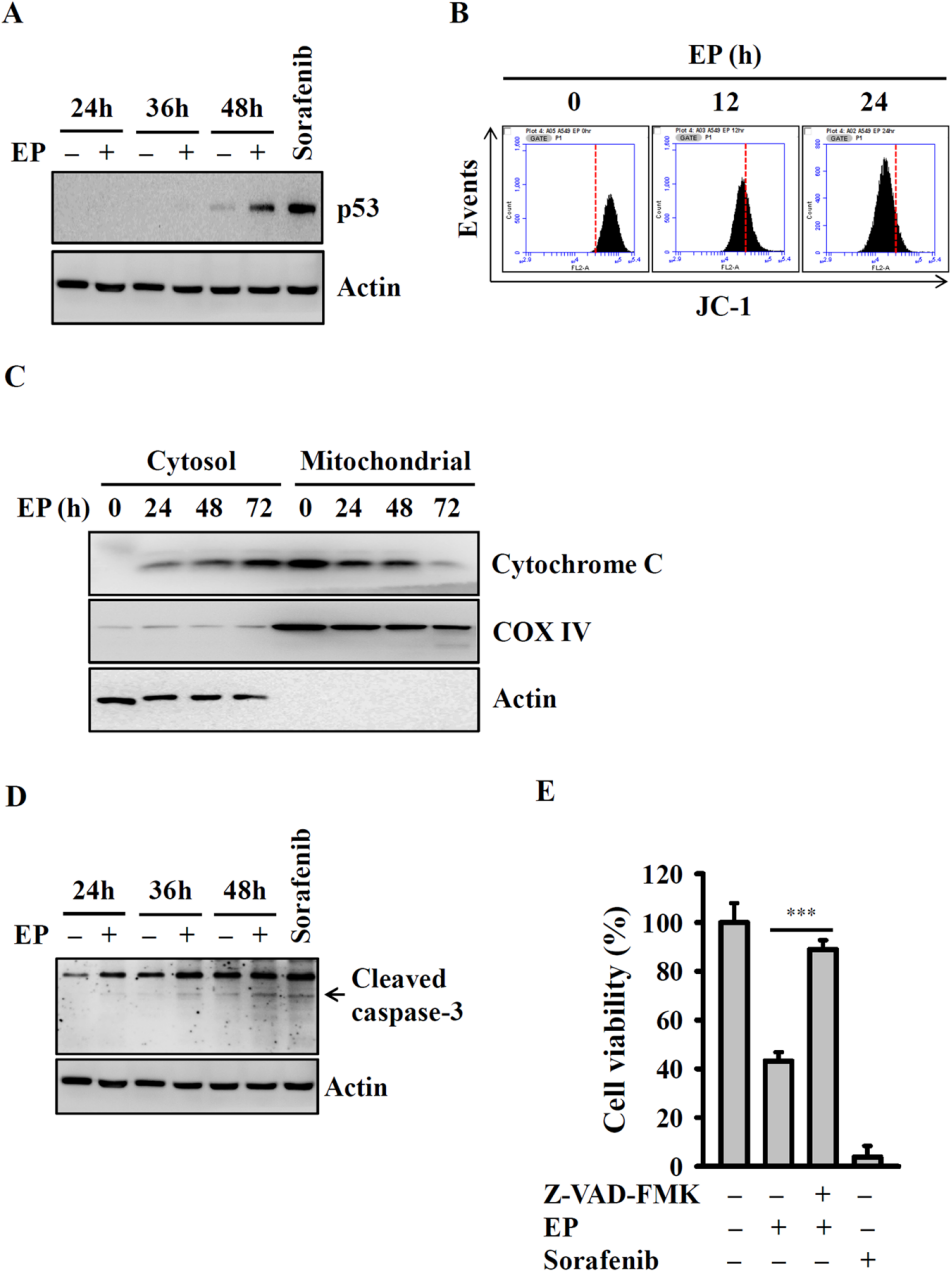
EP induced mitochondrial damage and caspase-dependent apoptosis. (**A**) The expression of p53 assayed by Western blot. (**B**) Mitochondria membrane potential assayed by JC-1 staining. (**C**) The cytochrome *c* releasing into cytosol assayed by Western blot. (**D**) The expression of cleaved caspase-3 assayed by Western blot. (**E**) Effect of pan caspase inhibitor (Z-VAD-FMK) on EP-mediated cytotoxicity. *** indicates significant differences at the levels of *p* < 0.001. The blots in (**A**,**C**,D) were cropped; full-length blots are included in Sup. Fig. 11.

To identify the molecular mechanism by which EP induced apoptosis, this study examined the levels of tumor suppressor gene p53 (p53) in EP treated A549 cells using Western blot^17^. We found that 20 µM of EP upregulated the p53 expression after 48 h treatment (Fig. 3A). Upregulation of p53 may lead to the mitochondrial damage^18^, which result in the apoptotic factors releasing, and then activate downstream apoptotic executor, caspase-3^17,18^. Therefore, we next examined the mitochondrial dysfunction by measuring mitochondrial membrane potential by flow cytometry. Our results showed that 20 µM of EP induced time-dependent (12 and 24 h), red shift (loss) in fluorescence intensity as determined using JC-1 lipophilic fluorochrome (Fig. 3B), indicated that EP affected the mitochondrial function of A549 cells. Next, the effect of EP on cytochrome *c* release was determined by Western blot. For this, the A549 cell lysates were sub-fractionated into cytosolic fraction and mitochondrial fraction. Western blot results demonstrated that there was no detectable amount of cytochrome *c* in the cytosolic fraction of untreated A549 cells. However, detectable cytochrome *c* release observed after 24 h of EP treatment and increased progressively up to 72 h, while there was a concomitant decreased mitochondrial cytochrome *c* level (Fig. 3C). Thus, our data demonstrated that in A549 cells EP-induced cytochrome *c* release from mitochondria to cytosol. Moreover, we also determined the effect of EP on caspase-3 activation in A549 cells, and found that EP treatment time-dependently increased the active form of caspase-3 (cleaved caspase-3) (Fig. 3D). To address the activation of caspase-3 required for the cell viability inhibition, A549 cells co-treated with caspase inhibitor and/or EP. The results showed that incubation with pan-caspase inhibitor (Z-VAD-FMK) (20 µM) significantly blocked the EP-induced cell viability inhibition in A549 cells (Fig. 3E).

### EP induced ROS-dependent apoptosis

**Figure 4:**
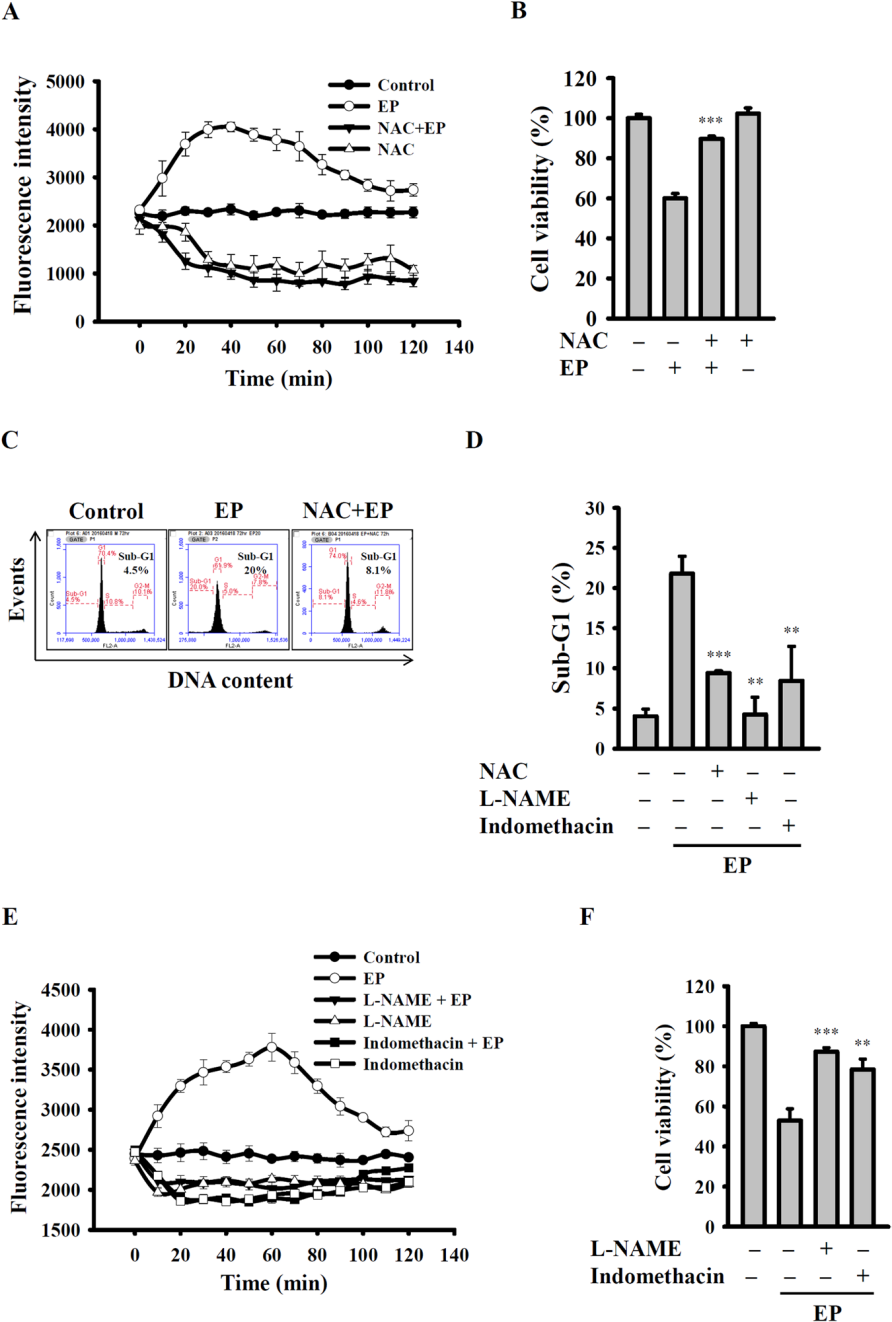
EP induced ROS-dependent apoptosis. ROS production assayed by H_2_DCFDA staining. (**B**) Effect of NAC on EP-mediated cytotoxicity. (**C**) Effect of NAC on EP-mediated sub-G1 phase increase. (**D**) Effect of L-NAME and indomethacin on EP-mediated sub-G1 phase increase. (**E**) Effect of L-NAME and indomethacin on EP-mediated ROS production. (**F**) Effect of L-NAME and indomethacin on EP-mediated cytotoxicity. ** and *** indicate significant differences at the levels of *p* < 0.01 and *p* < 0.001, respectively, compared to EP-treated cells.

It is previously demonstrate that ROS mediates intracellular signaling cascade and triggers programmed cell death pathways^19^. This study used H_2_DCFDA as a probe to examine intracellular ROS levels. The results showed that 20 µM of EP treatment time-dependently enhanced the mean DCF fluorescence intensity (increased ROS levels) in A549 cells, indicated that EP acted as a pro-oxidant (Fig. 4A). In contrast, the known antioxidant, *N*-acetylcysteine (NAC) reduced the intracellular ROS levels (Fig. 4A). To evaluate the effect of ROS in EP-induced cell death, A549 cells were treated in the presence or absence of NAC. The results showed that A549 cell death induced by 20 µM of EP significantly attenuated by the addition of NAC (Fig. 4B). Moreover, it was also observed that NAC enhanced the EP-treated A549 cell viability, which was accompanied by reduced sub-G1 phase cells (Fig. 4C), as well as reduced caspase-3 activation (Sup. Fig. 3A), DNA damage (Sup. Fig. 3B), cytosolic cytochrome *c* release (Sup. Fig. 3C) and MMP loss (Sup. Fig. 3D). Next, our study investigated whether ROS-generating enzymes involved in EP-mediated apoptosis. A549 cells were treated with EP in the presence or absence of various ROS generating enzymes inhibitors including NDGA (lipoxygenase inhibitor), L-NAME (iNOS inhibitor), allopurinol (xanthine oxidase inhibitor), indomethacin (cyclooxygenase inhibitor), rotenone (mitochondrial complex-I inhibitor), apocynin (NADPH oxidase inhibitor), or ketoconazole (cytochrome p450 inhibitor) for 30 min, and then the cells in sub-G1 phase was determined. The results showed that ROS generating enzymes inhibitors indomethacin and L-NAME reduced the EP-induced sub-G1 phase cell population (Fig. 4D), while the other enzymes inhibitors did not exhibited such effect (Sup. Fig. 3E). Further, it was also observed that EP-mediated ROS generation (Fig. 4E) and cell death (Fig. 4F) significantly attenuated by indomethacin and L-NAME.

### Autophagy presented EP-mediated A549 cell death

**Figure 5:**
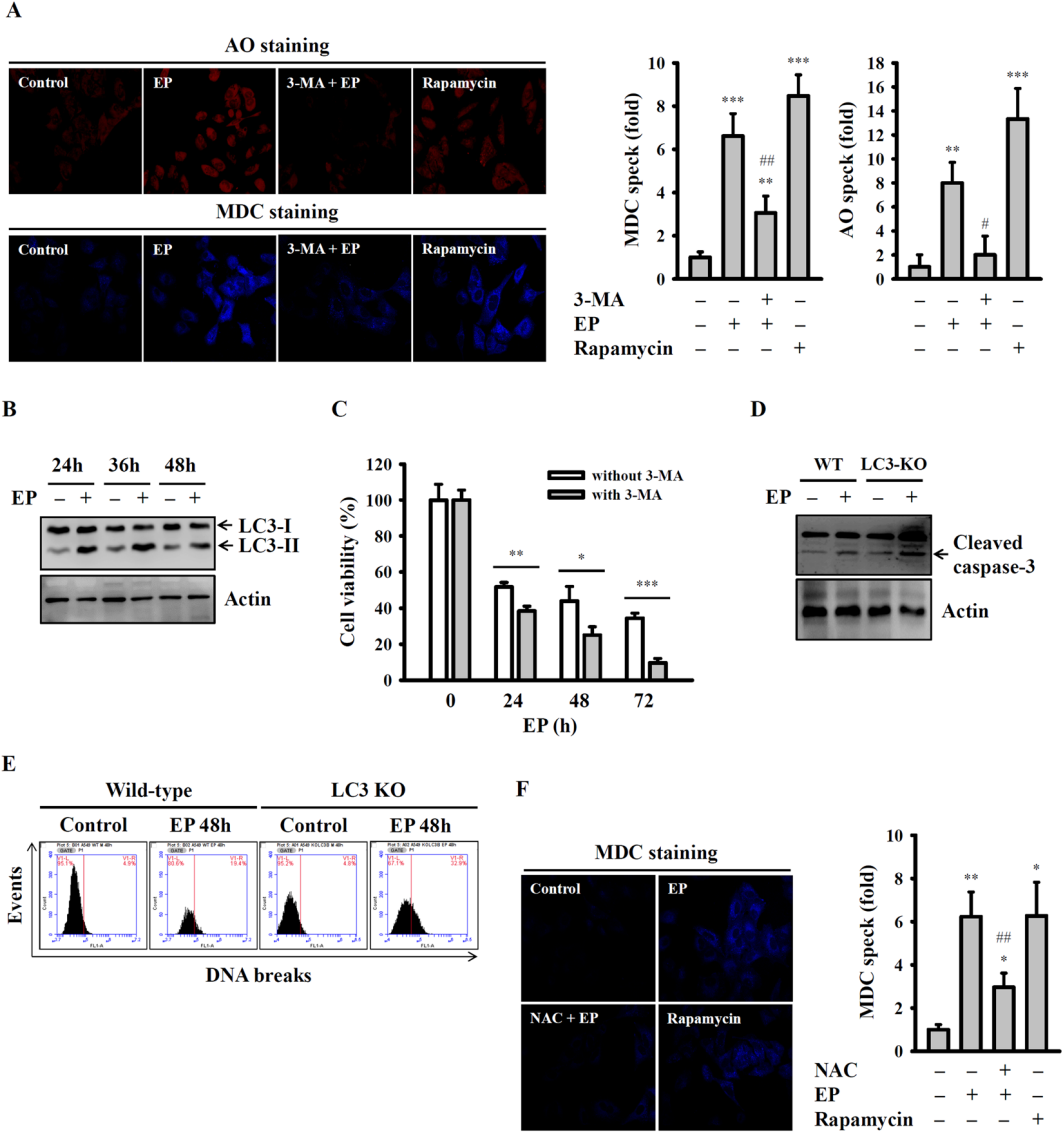
Autophagy inhibited EP-mediated cell death. (**A**) Effect of EP on autophagy induction assayed by AO and MDC staining. Qualitative assay differentiated by Image-J software. (**B**) The expression of LC-3 assayed by Western blot. (**C**) Effect of autophagy inhibitor 3-MA on EP-mediated cytotoxicity. (**D**) Effect of EP on caspase-3 activation in wild type and LC3 knockout A549 cells. (**E**) Effect of EP on DNA breaks in wild type and LC3 knockout A549 cells. (**F**) Effect of NAC on EP-mediated autophagy induction assayed by MDC staining. Qualitative assay differentiated by Image-J software. *^,^** and *** indicate significant differences at the levels of *p* < 0.05, *p* < 0.01 and *p* < 0.001, respectively, compared to control cells or as indicated. ^#^ and ^##^ indicate significant differences at the levels of *p* < 0.05 and *p* < 0.01, respectively, compared to EP-treated cells. The blots in (**B**,**D**) were cropped; full-length blots are included in Sup. Fig. 12.

It is previously demonstrated that autophagy is a process of protein recycling, and it can identify by the development of acidic vesicular organelles (AVOs). To detect the AVOs in EP-treated A549 cells, we used the lysosomotropic agent, acridine orange (AO), which protonated form accumulates in acidic compartments and emitted red fluorescence. Our flow cytometric analysis results showed that A549 cells without EP treatment exhibited green fluorescence (control). However, when A549 cells treated with 20 µM of EP for 24 h showed an increased red fluorescence (increase in acidity of AVOs), indicated an enhanced autophagy (Fig. 5A). As expected, A549 cells pre-incubated with an autophagy inhibitor, 3-MA (5 mM) for 30 min reduced the EP-induced red fluorescence, whereas 100 nM of rapamycin (autophagy inducer) treatment for 4 h increased the red fluorescence (Fig. 5A). Additionally, we also determined the EP-induced autophagy in A549 cells by monodansylcadaverine (MDC) staining method. Following incubation without or with 20 µM of EP for 24 h, we observed an increased fluorescent signal of MDC staining as compared with controls, whereas 3-MA abolished this effect (Fig. 5A). It is previously demonstrated that the hallmark of autophagy is the conversion of cytosolic LC3-I to the autophagosome-associate LC3-II. Therefore, we next determined the expression of LC3-II using Western blot. Our results showed that EP treatment time-dependently enhanced the LC3-II expression in A549 cells as compared with control untreated cells (Fig. 5B). It was interesting to note that in another human lung cancer cell lines CL1-1 and CL1-5, 20 µM of EP did not affect the expression of LC3-II (Sup. Fig. 4A), and the intensities AO and MDC signal (Sup. Fig. 4B), indicated that EP did not induce autophagy in these cell lines. Next, our study examined the role of autophagy in EP-induced A549 cell death. The results showed that EP time-dependently reduced the A549 cell viability, whereas this effect was increased by 3-MA, indicated that EP-induced autophagy to impair the sensitivity of A549 cells. The protective effect of autophagy on A549 cell death was further confirmed by EP-mediated MMP loss (Sup. Fig. 5A) and cells in sub-G1 phase (Sup. Fig. 5B), which was increased by 3-MA. To provide the direct evidence for the effect of autophagy on EP-mediated cell death, LC3 expression in A549 cells was knockdown by Crispr-Cas9 system (Sup. Fig. 5C). We found that EP-mediated caspase-3 activation (Fig. 5D), DNA breaks (Fig. 5E), cytochrome *c* release (Sup. Fig. 5D) and the cells in sub-G1 phase (Sup. Fig. 5E) increased in LC3 knockdown cells, as compared the wild-type cells. Furthermore, we also found that EP-mediated increase in fluorescent signal of MDC (Fig. 5F) and LC3-II expression (Sup. Fig. 5F) were reduced by NAC. These results indicated that EP-induced autophagy regulated by ROS. Interestingly, although 3-MA enhanced the cytotoxicity of EP, the cell viability was significantly increased by caspase inhibitor Z-VAD-FMK in 3-MA/EP-treated A549 cells (Sup. Fig. 5G).

### Ep inhibited proliferation and migration of A549 cells by attenuated NLRP3 inflammasome

**Figure 6:**
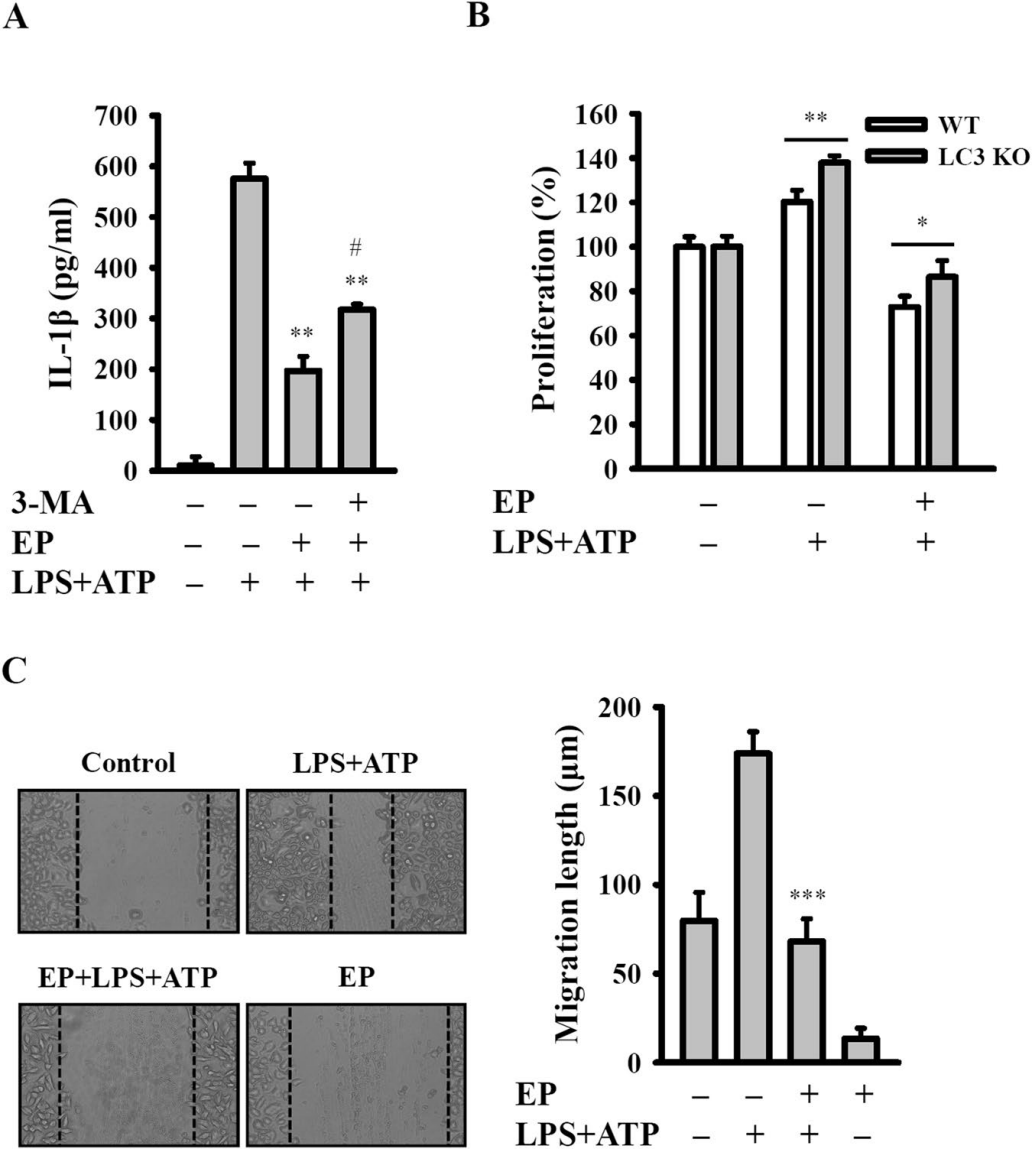
EP inhibited proliferation and migration of A549 cells by inhibition of NLRP3 inflammasome. (**A**) The expression of IL-1β assayed by ELISA. (**B**) Effect of EP on cell proliferation in LPS + ATP-treated wild type and LC3 knockout A549 cells. (**C**) Effect of EP on migration ability in LPS + ATP-treated A549 cells. *^,^ ** and *** indicate significant differences at the levels of *p* < 0.05, *p* < 0.01 and *p* < 0.001, respectively, compared to LPS + ATP-treated cells or as indicated. ^#^ indicates significant differences at the levels of *p* < 0.05 compared to EP/LPS + ATP-treated cells.

Previous studies indicate that NLRP3 inflammasome exerts diverse and sometimes contrasting roles in the development of different cancers including lung cancer^14^. We next investigated the involvement of NLRP3 inflammasome in EP-induced A549 cell death. To do this, A549 cells were treated with the known NLRP3 inflammasome activators LPS and ATP. Our results showed that activated NLRP3 inflammasome was attenuated by treatment with 20 µM of EP as evidenced by decreased IL-1β secretion (a known NLRP3 inflammasome end-product) (Fig. 6A). However, the autophagy inhibitor 3-MA partially reversed this effect (Fig. 6A). Additionally, it was also observed that elevated NLRP3 inflammasome promoted the proliferation of A549 cells; however, EP abolished such effect (Fig. 6B). Interestingly, LPS/ATP-induced proliferation of A549 cells increased in LC3 knockdown cells compared with wild type cells (Fig. 6B). Activated NLRP3 inflammasome significantly promoted the A549 cell migration, which reversed after EP treatment, determined by wound healing assay (Fig. 6C). Furthermore, this study found that EP attenuated the migration of CL1-1 and CL1-5 (Sup. Fig. 6A). Next, we examined whether or not NLRP3 inflammasome was activated in CL1-1 and CL1-5 cells. The results showed NLRP3 inflammasome was not activated in CL1-1 and CL1-5 cells, as evidenced by there was no significant IL-1β secretion from the LPS/ATP-treated CL1-1 and CL1-5 cells (<5 pg/ml) (Sup. Fig. 6B).

### EP treatment affected the signaling protein expression

**Figure 7:**
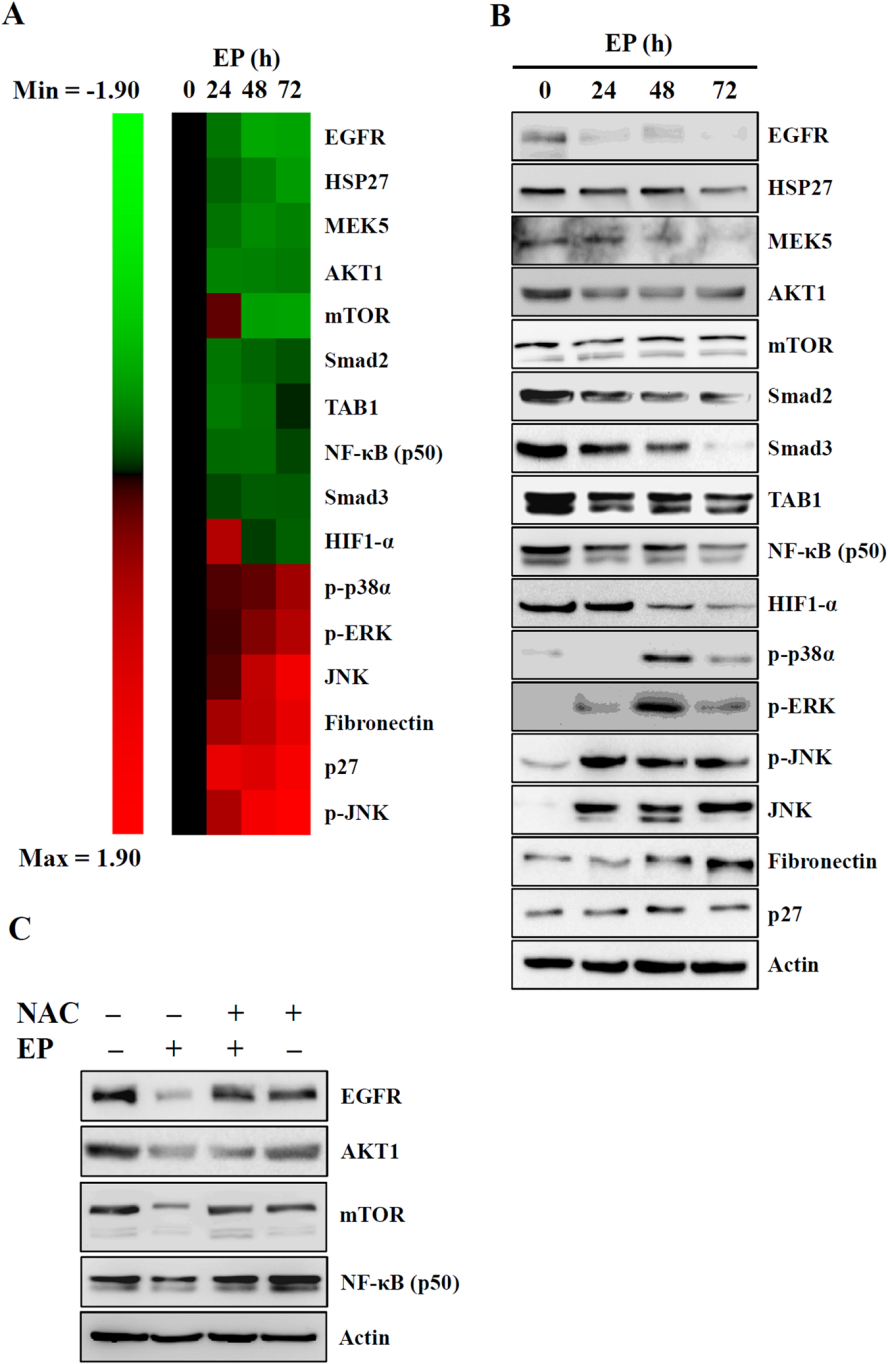
EP affected the expression of the proteins regulating cell proliferation. (**A**) The protein expression levels presented by heat-map. Green color indicated decrease of protein expression and red color indicated increase of protein expression after EP treatment. (**B**) The protein expression levels confirmed by Western blot. (**C**) Effect of NAC on EP-mediated changes in protein expression. The blots in (**B**,**C**) were cropped; full-length blots are included in Sup. Fig. 13.

The aforementioned results of this study demonstrated that EP treatment attenuated the A549 cell viability and induced apoptosis. We then investigated the effect of EP on the expression of apoptosis signaling proteins using Micro-Western Array (MWA) assay. The protein expression profile determined by MWA in A549 cells treated with 20 µM of EP for 0, 24, 48, and 72 h, with 192 different antibodies (Sup. Fig. 7). The results showed that EP treatment significantly decreased the proteins level of EGFR, HSP27, MEK5, AKT1, Mtor, Smad2, TAB1, NF-κB (p105/p50), Smad3, and HIF1-α. Additionally, EP treatment significantly increased the protein levels of p-p38α, p-ERK1/2, JNK, fibronectin, p27 and p-JNK (Fig. 7A). On the other hand, the expression level of apoptosis proteins in EP-treated A549 cells was also determined by conventional Western blot (Fig. 7B). Consistent with MWA data, western blot results showed that EP treatment to A549 cells affected the proteins involved in regulating cell cycle, proliferation, survival, DNA damage and mTOR signaling pathways. In particular, 20 µM of EP treatment resulted the reduced protein expression levels of EGFR, MEK5, AKT1, Smad2, Smad3, TAB1, NF-κB (p105/p50) and HIF1-α, while increased the expression of p-p38α, p-ERK1/2, JNK, fibronectin, p27 and p-JNK (Fig. 7B). Our previous data of this study indicated that ROS played an important role in EP-mediated A549 cell death, caspase-3 activation and DNA damage (Fig. 4 and Sup. Fig. 3). Herewith we found that EP treatment down regulated the protein expression of EGFR, AKT1, mTOR, and NF-κB (p105/p50), which abolished by ROS scavenger NAC (Fig. 7C), indicated that EP-induced ROS played an important role in regulating these apoptotic protein expression.

### The synergistic effect of EP and Sorafenib

The synergistic effect of EP combined with Sorafenib (Raf kinase inhibitor in multiple cancer cells) in A549 cells was examined by following the previously described experimental procedure^20^. The inhibition rate of EP (5, 10 and 20 µM) combined with Sorafenib at different concentrations (2.5, 5 and 10 µM) on the growth of A549 cells was shown in Table 2. We found that the combination index (CI) value at 10 µM of EP combined with 2.5 µM of Sorafenib was 1.30, which indicated a better synergistic effect on growth inhibition of A549 cells than individual compounds. These results indicated the EP might enhance the anticancer drug Sorafenib effect in clinical lung cancer therapy.

**Table 2:** EP and Sorafenib Drug Combination Effect.

Sora + EP	2.5 + 10 μM	5 + 10 μM	10 + 10 μM	2.5 + 5 μM	2.5 + 20 μM
Viability	76 ± 10.8%	63 ± 13.5%	49 ± 13.1%	73 ± 15.3%	47 ± 8.4%
Cal. Viability for Sora	6.95 μM	11.67 μM	16.85 μM	7.97 μM	17.85 μM
Cal. Viability for EP	10.67 μM	11.06 μM	24.01 μM	12.06 μM	25.40 μM
*CI value	1.30	0.85	0.56	0.73	0.93

*CI = 1.00, additive; CI < 1.00, synergistic; CI > 1.00, antagonistic.

## Discussion

Lung cancer is the most common prevalent type of cancer in the world. Owing to several limitations associated with early stage diagnosis, majority of lung cancer patients diagnosed at the median or advanced stage. Additionally, due to the moderate progress provided by chemotherapeutics and the development of drug resistance^1,2^, the use of natural products for lung cancer treatment has attracted the attention^5^. In this study, chemical examination of *Phoma sp*. resulted in the isolation of three steroidal compounds, ergosterol, EP, and DEP. The three isolated metabolites showed tumor specific varied cytotoxic effect in four types of cancer cells screened, with EP being more sensitive to lung cancer cell line A549 cells. Therefore, this study demonstrated the action mechanism of EP in the survival and apoptotic pathways in A549 cells. In this connection, it is interested to note that the chemical structure of EP contained endoperoxide functional group similar to artemisinin. It is known that artemisinin is currently the frontline treatment for malaria and it has recently demonstrated as an anticancer drug^21,22^. The antimicrobial and antitumor potential of EP described previously^19,23,24^; however, there is no report addressing the growth inhibitory action mechanism of EP against A549 cells.

The results of this study demonstrated that EP attenuated the viability of cancer cells belongs to different cancer types (Table 1). Next, our study investigated that the action mechanism of EP inhibited A549 cell viability. It is known that apoptosis is a well-characterized programmed cell death, which plays a critical role in the development and homeostatic maintenance of living cells. Death cells are distinguish by unique morphological and biochemical characteristics^17^. In this study, the results showed that EP treatment time-dependently increased the population of sub-G1 cells, cell apoptosis, DNA fragmentation, while reduced the colony formation ability of A549 cells (Fig. 2). This data demonstrated that EP induced A549 cell death through apoptotic pathway. It is known that cell death followed two main apoptotic pathways including extrinsic (death receptor) and the mitochondria-dependent intrinsic^10^. Previous reports demonstrated that depolarization of mitochondrial membrane activated the release of pro-apoptotic factors such as cytochrome *c* into the cytosol^18^. Therefore, we examined the involvement of mitochondria in EP-induced A549 cell apoptosis. On the other hand, the tumor-suppressor gene p53 is widely known for its role in cell differentiation, cell cycle regulation and apoptosis in response to DNA damage^25,26^. p53 is a short lived protein and in normal physiological conditions it appears at low level, however its level becomes increase in response to DNA damage^25,26^. Our results showed that EP induced mitochondria-dependent intrinsic apoptosis in A549 cells, as evidenced by increased p53 expression, cleaved caspase-3, and reduced mitochondrial membrane potential and cytochrome *c* release (Fig. 3).

ROS is a collective term, which refers unstable, reactive, partially reduced oxygen derivatives that involve in the metabolic processes^27^. A low level of ROS is required for the regulation of cellular signaling and gene expression. However, the role of ROS in cancer cells is complicated and may play opposite role in a variety of pathophysiological conditions^28^. It is known that ROS can generate intracellularly from dysregulated mitochondria^29^, and high level ROS can cause the oxidative stress and damage the cellular components including lipids and DNA, and induce cell growth inhibition and apoptosis^28^. In contrary, it previously demonstrates that anticancer drugs promoted the ROS production in cancer cells and induced apoptosis^30^. Here, our results showed that EP treatment increased the ROS production in A549 cells (Fig. 4A). Further, our data showed that EP induced ROS production enhanced the A549 cells apoptosis; however, an antioxidant agent NAC reduced the levels of A549 cells in sub-G1 phase, DNA damage, loss of mitochondrial membrane potential (Fig. 4). The pro-apoptotic effects of EP induced ROS were in agreement with previous reports^31–34^. On the other hand, although ROS usually promotes apoptosis, however, previous reports indicate it may inhibit apoptosis depending on the type of cancer as well as cell line culture conditions^35–37^. The known ROS-producing enzymes in mammalian cells are NADPH oxidase, xanthine oxidase, lipoxygenases and cytochrome P450^38^. Here, our results showed that EP-induced ROS generation and apoptosis attenuated by indomethacin, a known cyclooxygenase inhibitor. Thus, our results were in parallel with previous reports that indomethacin induce apoptosis in A549 cells by mitochondrial damage^29^. Furthermore, our results were in agreement with earlier reports that EP-induced A549 cell death abolished by L-NAME, an iNOS inhibitor; as well as indomethacin (Fig. 4F)^39^. Taken together, our results demonstrated that EP-induced ROS generation in A549 cells were attenuated by antioxidant NAC and by the inhibitors of cyclooxygenase and iNOS. It was interested to note that the peroxide group of EP was important for ROS production and to induce apoptosis in A549 cells, as evidenced by ergosterol (lacking peroxide group) did not induce ROS production (Sup. Fig. 8A), and induce less percentage of A549 cells in the sub-G1 phase than EP (Sup. Fig. 8B).

Autophagy is a highly in conserved in intracellular degradative process. It plays contextual functions in the cancer cells, as it kills cancer cells but also protects cancer cells against injury^40,41^. A hallmark of autophagy is the formation of characteristic AVOs in autophagy sequester through cytoplasmic proteins. It demonstrates that AO moves freely across biological membrane and accumulates in acidic organelles in a pH-dependent manner, and is commonly used to identify AVOs. Under AO staining, the nucleus and cytoplasm fluorescence green, whereas the acidic compartments fluorescence is bright red with blue light excitation. Our results showed that EP treated A549 cells exhibited an enhanced red fluorescence similar to that of an autophagy enhancer, rapamycin, indicated an increased autophagy. In contrast, the autophagy inhibitor 3-MA abolished such effect (Fig. 5A). Moreover, it was also observed that the enhanced MDC staining and AO speck formation in EP-treated A549 cells, supported the autophagy induction in EP-treated A549 cells (Fig. 5A). It is known that autophagy-related genes LC3-I can convert into LC3-II during autophagy, and participate in autophagosome formation^40^. Thus LC3 consider as an important maker of autophagosome, and the conversion of LC3-I to LC3-II correlate with the extent of autophagy. In this study, the western blot analysis data showed that EP treatment enhanced the LC3-II expression in A549 cells (Fig. 5B). Additionally, when A549 cells treated with the autophagy inhibitor 3-MA or EP resulted in the reduced cell viability, and increased expression of cleaved caspase-3, suggested that autophagy serves as a protective role (Fig. 5C,D). Thus, our results demonstrated that EP induced apoptosis and autophagy in A549 cells. These results were in parallel with previous reports that increased autophagy promotes apoptosis in various types of cancer cell lines^41,42^, in contrast to reduced autophagy increased apoptosis^43,44^. Several signaling pathways were reported to involve in the autophagy induction. This study observed that ROS production played an important role in the EP-induced apoptosis and autophagy in A549 cells, which were coincide with the previous reports in human non-small cell lung cancer and hepatoblastoma cells^42–45^. Activation of p38 leads to the autophagy induction in cathepsin S inhibited human oral cancer cells^46^, and in interferon regulatory factor-1 activated mice^47^. It is previously demonstrated that in human lung cancer cells, the signaling pathways JNK and ERK play important roles in autophagy induction^43–48^. The results of this study showed that EP treatment enhanced the phosphorylation levels of ERK, JNK and p38 in A549 cells, however, the role of these signaling pathways in EP-mediated autophagy induction need further investigation.

**Figure 8:**
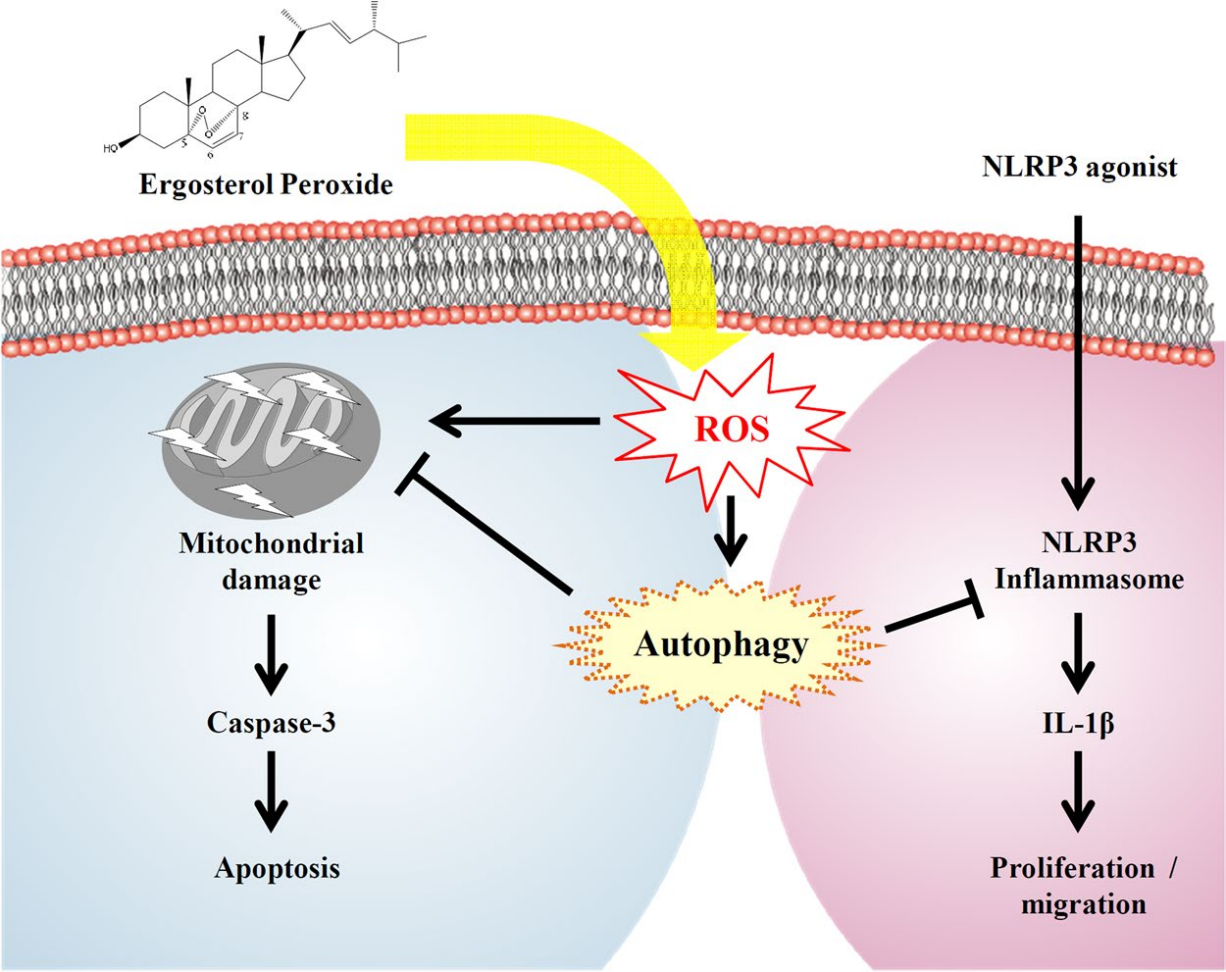
Schematic of the representation of EP effect in A549 cells.

Our MWA results showed that EP treatment affected the protein expression of lung cancer cells apoptosis (Fig. 7). For example, EP treatment reduced the expression levels of EGFR and AKT, which are important signaling axis for the cell growth of A549 cells^49^. It is demonstrated that the elevated HIF-1 protein expression is associated with tumor growth and metastasis, and considered as a therapeutic target^50^. Our results showed that EP reduced the expression of HIF1-α, indicated its role in A549 cell growth inhibition. Previous reports showed that increased HSP27 expression in human lung cancers was associated with chemotherapy resistance^51,52^. We found that HSP27 was expressed in A549 cells and attenuated by EP treatment, indicated a potential role of EP in HSP27-targeted lung cancer^53^. It is previously demonstrated that the expression level of TAB1 in non-small cells lung carcinoma tissue was significantly higher than that in tumor-adjacent normal tissue, and the TAB1 expression level was negatively related to patient prognosis^54^. In this study, the results showed that EP treatment attenuated the TAB1 expression in A549 cells. Furthermore, we also observed that EP treatment resulted in the reduced protein expression of Smad2 and Smad3, which are important for lung cancer growth and metastasis^55,56^. NLRP3 inflammasome is a protein complex, which increased the secretion of various inflammatory components including IL-1β^57^. Additionally, previous reports indicate that elevated NLRP3 inflammasome promotes the proliferation and migration of A549 cells^14^, but inhibits tumorigenesis in colitis-associated cancer^58^. The results of this study showed that EP attenuated the elevated NLRP3 inflammasome activation through reduced IL-1β secretion, in addition to decreased proliferation and migration of A549 cells (Fig. 6). As EP induced both apoptosis and autophagy, we speculated that autophagy induction was the protective mechanism in response to the EP-mediated apoptosis. Based on our results, it was concluded that the induction of autophagy decreased the apoptosis inducing activity of EP. We also observed that inhibition of autophagy enhanced the cell proliferation in NLRP3 inflammasome-activated A549 cells. Thus, combination of EP, autophagy inhibitor and NLRP3 inflammasome inhibitor will be a potential strategy in the clinical application. A schematic representation of the present study was presented in Fig. 8.

## Conclusion

This study demonstrated that the marine fungus, *Phoma sp*., compound ergosterol peroxide (EP) exhibited potential growth inhibitory properties against lung cancer cells through induction of mitochondria dependent apoptosis and autophagy. MWA approach might be a useful tool for rapid identification of potential small molecule on protein signaling networks. Further, inhibition of enhanced NLRP3 inflammasome by EP might be a new therapeutic candidate for treating inflammation-promoted cell growth and migration of human lung cancer cells. However, further studies were required to address the effect of EP against other human lung-cancer cell lines as well as its role in other underlying mechanisms. Additionally, further studies using *in vivo* experimental models were also warrant.

## Methods

### Fungus identification

The marine fungus collected from 400 m depth of Bering Sea and taxonomically identified as *Phoma sp*. by gene identification (Sup. Fig. 9). A voucher specimen (No. 1222) was deposited in Institute of Biological Chemistry, Academia Sinica, Taiwan.

### Extraction and isolation of *Phoma sp*. isolates

The fungus *Phoma sp*. grown at room temperature (25 ± 2 °C) in Petri dishes containing 20 ml of potato dextrose agar (PDA) for 21 days. The mycelium was collected after 21 days cultivation by filtration, and extracted with ethyl acetate (EtOAc) for 24 h. Extraction repeated for five times. The combined EtOAc extracts were filtered, and then concentrated in a rotary evaporator under at 40 °C and 60 rpm to get crude extract. The crude extract was subjected to size-exclusion chromatography using Sephadex LH-20 to yield four fractions A, B, C and D. Fraction B was separated by reverse-phase HPLC (Agilent RP-HPLC system, C-18 discovery column 250 × 10 mm equipped with guard column). The HPLC conditions as follows: injection volume: 20 µl at 2 mg/ml in methanol (MeOH). The flow rate was set at 1.0 ml/min and the detection wavelength monitored at 230 and 254 nm. The mobile phase contained the gradient of MeOH/H_2_O/acetic acid (75:24.9:0.1) for 20 min, followed by 30 min elution with 100% MeOH. The chemical structures of ergosterol, ergosterol peroxide (EP) and 9,11-dehydroergosterol peroxide (DEP) were identified with the aid of their spectral data (NMR and Mass), which were identical with literature values (Sup. Fig. 10).

### Reagents and cell culture

Human lung cancer line A549, human liver cancer line J5, human cervical cancer line HeLa, mice macrophages RAW 264.7, and human normal lung cells Beas-2b were maintained in RPMI-1640 medium (CORNING, Mediatech, Inc.) supplemented with 10% fetal bovine serum (FBS). Human breast cancer line MCF-7 cells were maintain in DMEM medium (CORNING, Mediatech, Inc.), supplemented with 10% FBS containing with 0.005 mg/ml Bovine insulin. Cultures were grown in a humidified incubator at 37 °C and 5% CO_2_.

### Cell proliferation assay

Cell viability was assessed by MTT assay following the previously reported procedures^59^. Cells were seeded into 96-well plate at a density of 8,000 cells/well and stabilized at 37 °C in 5% CO_2_ for 24 h. Cells were incubated for 24 h with ergosterol, EP, and DEP. Then cells were treated with 20 µl of MTT solution (0.5 mg/ml) for another 5 h. The formazan crystals dissolved in 100 µl of DMSO. Cell viability assessed by measuring the absorbance at 570 nm wavelength using an EMax Microplate Reader (Molecular Devices, Sunnyvale, CA, USA). To investigate the effect of NLRP3 inflammasome in the cell proliferation, cells were pre-treated with 20 µM of EP for 30 min and then stimulated with 1 µg/ml of LPS for a period of 8 h with 5 mM ATP for the last 30 min. The cell proliferation was measured by MTT assay after 16 h of ATP treatment. For the positive control, cells were incubated for 24 h with 10 µM of Sorafenib.

### Cell colony and migration assay

A549 cells were cultured for 24 h in a six-well plate at a density of 80 cells per well, then different doses (1, 5, or 20 µM) of EP was added into the wells. The cultures were grown for 3 days. In day 4, culture medium was replaced with a fresh medium containing the same doses of EP and continued the cultivation until day 7. The colonies fixed in a 4% ice-cold paraformaldehyde for 15 min at 37 °C, and each well stained with 0.1% crystal violet overnight at room temperature. The colonies then counted. Cell migration was measured by scratch assay. Cells seeded in six-well plates until the cells reached to 100% confluence forming a monolayer, a sterile 200-µl pipette tip used to create a scratched clear zone on culture dish. A549 cells were incubated with 20 µM of EP for 30 min, and then treated with 1 µg/ml of LPS for 8 h with 5 mM ATP for the last 30 min. The wounds photographed at baseline (before EP treatment) and 16 h after ATP treatment, using a phase contrast microscope (CKX53, Olympus).

### ELISA assay

A549 cells were seeded in 24-well plates overnight. To measure IL-1β secretion^14^, cells were pretreated for 30 min with 20 µM of EP in the presence of 3-MA (5 mM), followed by 1 µg/ml of LPS for 8 h with or without 5 mM of ATP for the last 30 min. The cell supernatants were collected and centrifuged at 300 g for 8 min at 4 °C for elimination of dead cells. Next, supernatants were concentrated by centrifugation at 12,000 g for 30 min at 4 °C through a centrifuge tube with a cut-off of 10 kDa (Vivaspin 6; GE Healthcare, USA). The IL-1β level was measured by human ELISA kits from Affymetrix (eBioscience, Thermo Fisher Scientific Inc., MA, USA), according to the manufactures instructions.

### Autophagy staining

A549 cells were seeded at a density of 1 × 10^5^ cells/well in 12-wells plate overnight. Cells were incubated with or without 5 mM of 3-MA for 30 min, followed by with or without 20 µM of EP for 24 h. Cells incubated with 100 nM of rapamycin for 4 h as a positive control for autophagy induction. Next cells were stained with MDC (50 µM) or AO (1 µg/ml) for 30 min at 37 °C, and then they were washed with PBS three times, fixed with 4% paraformaldehyde for 30 min and washed with H_2_O for three times. Inverted confocal microscopy (Fluoview FV1000, Olympus, Japan) used to observe the change in autophagic vacuoles to capture images.

### Determination of A549 cells in Sub-G1 phase, apoptosis, DNA breaks and mitochondrial membrane potential

A549 cells were incubated with 20 µM of EP for the time as indicated. The cells in Sub-G1 phase were measured by flow cytometry after PI (10 µg/ml) staining. The apoptotic cells were measured by flow cytometry after Annexin V/PI staining (Abcam, USA). The DNA breaks were measured by Apo-direct kit (BD Bioscience, USA). The mitochondrial membrane potential was measured by flow cytometry after JC-1 staining (Thermo Fisher Scientific Inc., MA, USA). The cytochrome *c* release from mitochondria into cytosol was determined by detection of cytochrome *c* in the cytosolic and mitochondrial fractions using Western blot. The process of mitochondria isolation was followed by the protocol of Mitochondria isolation kit (Ab100170, Abcam, USA).

### Knockout of LC3β gene by CRISPR/Cas9-mediated genome editing

A549 cells were cultured in six-well dishes overnight. Following the protocol from the Protocol data-sheet of Santa Cruz Company, we added 1 µg per well of human LC3β KO DNA plasmid pool (sc-417828, Santa Cruz) and 1 µg per well of human LC3β HDR plasmid (sc-417828-HDR, Santa Cruz). After transfection, these cells were treated with 2 µg/ml of puromycin for 3 days. Surviving cells reseeded at 1 × 10^5^ cell of 6 cm dish for isolation of single cell clones. The knockout LC3β A549 was confirmed by classical western blot (Sup. Fig. 6C).

### ROS fluorescence assay

A549 cells were seeded at a density of 8,000/well in 96-wells plate. After incubation for overnight, culture medium replaced with PBS buffer, cells were stained with 2 µM of H_2_DCFDA for 30 min, followed by incubated for 30 min with 10 mM of NAC, 0.5 mM of L-NAME or 50 µM of indomethacin. Cells were then incubated with 20 µM of EP for 0–120 min. The method of detecting intracellular ROS was described previously for monitoring the fluorescence intensity of 2′,7′-dichlorofluorescein, the oxidation product of H_2_DCFDA^60^.

### Synergistic effect

The synergistic effects of EP combined with Sorafenib on A549 cells was studied following the previously described experimental procedure^20^.

### Micro-Western Array

A549 cells were treated with 20 µM of EP for different time points 0, 24, 48 and 72 h. Cells lysis was done following the previously reported method^61^. Scanned images saved as 16-bit tiff files for blotting data analysis. Genepix 8.0 (Molecular Devices) was used to record the mean by drawing an equally sized circle around the appropriately band for each analytical target. The background fluorescence recorded by placing an equal sized circle in the blank space to the left of the first sample. The intensity calculated by computing the channel 800 green color intensity. To normalize sample concentration, the intensities divided by β-actin calculated separately for each array print. Fold change calculated as the ratio of normalized intensity to the net intensity at the 0 h time point, minus one.

### Statistical analysis

Data presented as mean ± SD of minimum three independent experiments or representative experiments repeated more than three times. Quantitative data presented as mean ± SD. Students t-test used to determine the significance of difference between two groups. A probability of 0.05 or less considered statistically significant.

## Electronic supplementary material

Supplementary information
