# Supplementary material for: Ergosterol peroxide from marine fungus Phoma sp. induces ROS-dependent apoptosis and autophagy in human lung adenocarcinoma cells
Source: Sci Rep. 2018 Dec 18;8:17956. doi: 10.1038/s41598-018-36411-2 (PMC6298985; doi:10.1038/s41598-018-36411-2)
Supplement: Supplementary file 1 — Supplementary information [file 41598_2018_36411_MOESM1_ESM.docx]

Supplementary data for

**Ergosterol peroxide from marine fungus *Phoma sp.* induces ROS-dependent apoptosis and autophagy in human lung adenocarcinoma cells**

Han-Ying Wu^1,2,3^, Feng-Ling Yang^1^, Lan-Hui Li^4^, [Yerra Koteswara Rao](https://www.ncbi.nlm.nih.gov/pubmed/?term=Rao%20YK%5BAuthor%5D&cauthor=true&cauthor_uid=23431343)^5^, Tz-Chuen Ju^6^, Wei-Ting Wong^7^, Chih-Yu Hsieh^5^, Michael V. Pivkin^8^, Kuo-Feng Hua^5,9,10,*^, Shih-Hsiung Wu^1,*^

^1^Institute of Biological Chemistry, Academia Sinica, Taipei, Taiwan

^2^Institute of Biological Chemistry, Chemical Biology and Molecular Biophysics Program, Taiwan International Graduate Program, Academia Sinica, Taipei, Taiwan

^3^Department of Chemistry, National Tsing Hua University, Hsinchu, Taiwan

^4^Department of Laboratory Medicine, Linsen, Chinese Medicine and Kunming Branch, Taipei City Hospital, Taipei, Taiwan

^5^Department of Biotechnology and Animal Science, National Ilan University, Ilan, Taiwan

^6^Department of Animal Science and Biotechnology, Tunghai University, Taichung, Taiwan

^7^Graduate Institute of Life Sciences, National Defense Medical Center, Taipei, Taiwan

^8^G.B. Elyakov Pacific Institute of Bioorganic Chemistry FEB RAS, Vladivostok, Russia

^9^Department of Pathology, Tri-Service General Hospital, National Defense Medical Center, Taipei, Taiwan

^10^Department of Medical Research, China Medical University Hospital, Taichung, Taiwan

^*^Corresponding author: E-mail: kfhua@niu.edu.tw; Tel.: +886-(03) 931-7626 Fax: +886-(03) 931-1526; shwu@gate.sinica.edu.tw; Tel.: +886-(02) 2785-5696 Fax: +886-(02) 2653-9142


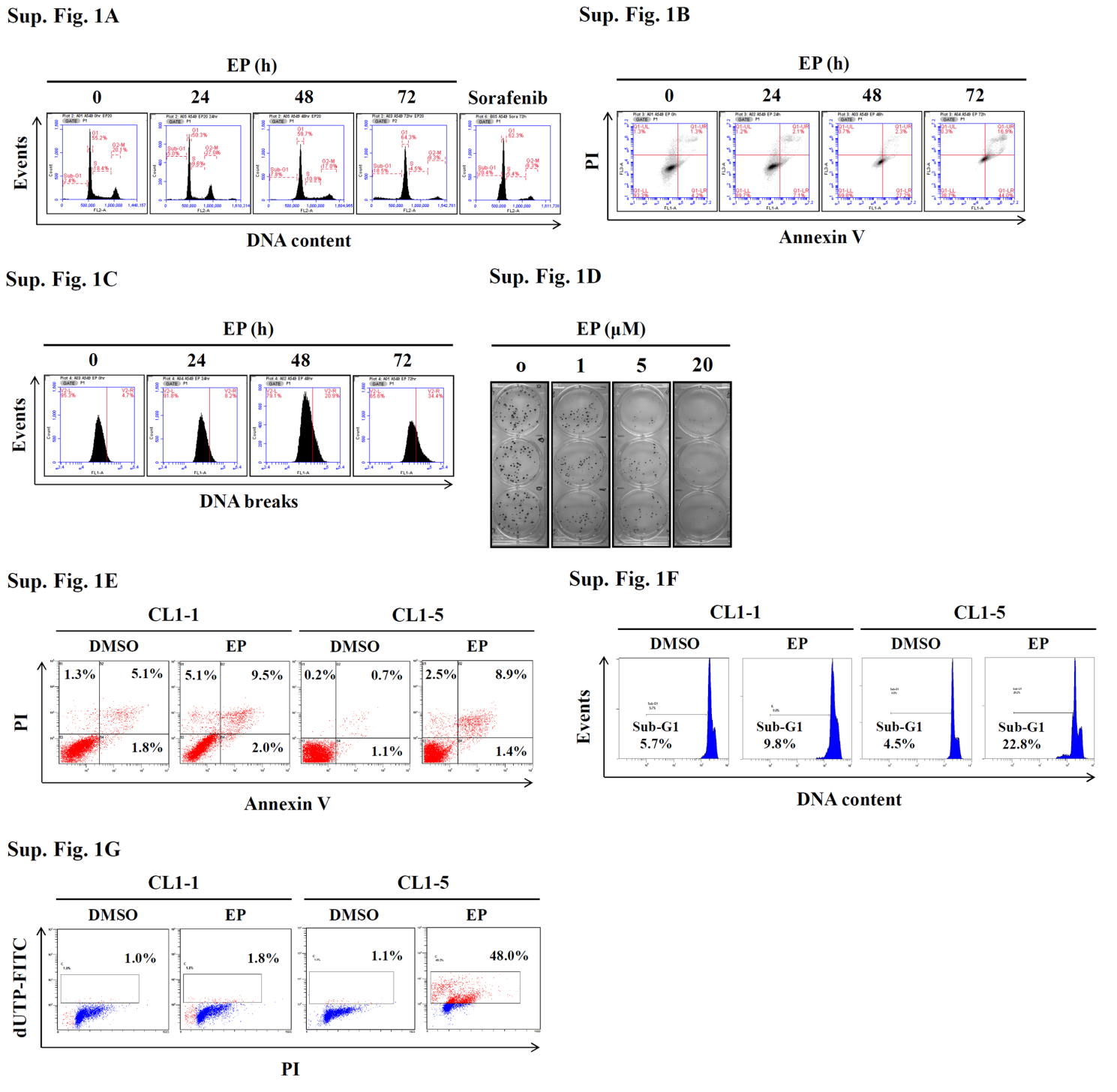


**Figure S1. EP induced apoptosis and DNA damage in A549.** (A) Cells in sub-G1 phase were assayed by PI staining in A549 cells. (B) Apoptosis assayed by PI and Annexin V double staining in A549 cells. (C) DNA breaks assayed by TUNEL assay in A549 cells. (D) Colony formation assay in A549 cells. (E) Apoptosis assayed by PI and Annexin V double staining in CL1-1 and CL1-5 cells. (F) Cells in sub-G1 phase were assayed by PI staining in CL1-1 and CL1-5 cells. (G) DNA breaks assayed by TUNEL assay in CL1-1 and CL1-5 cells.


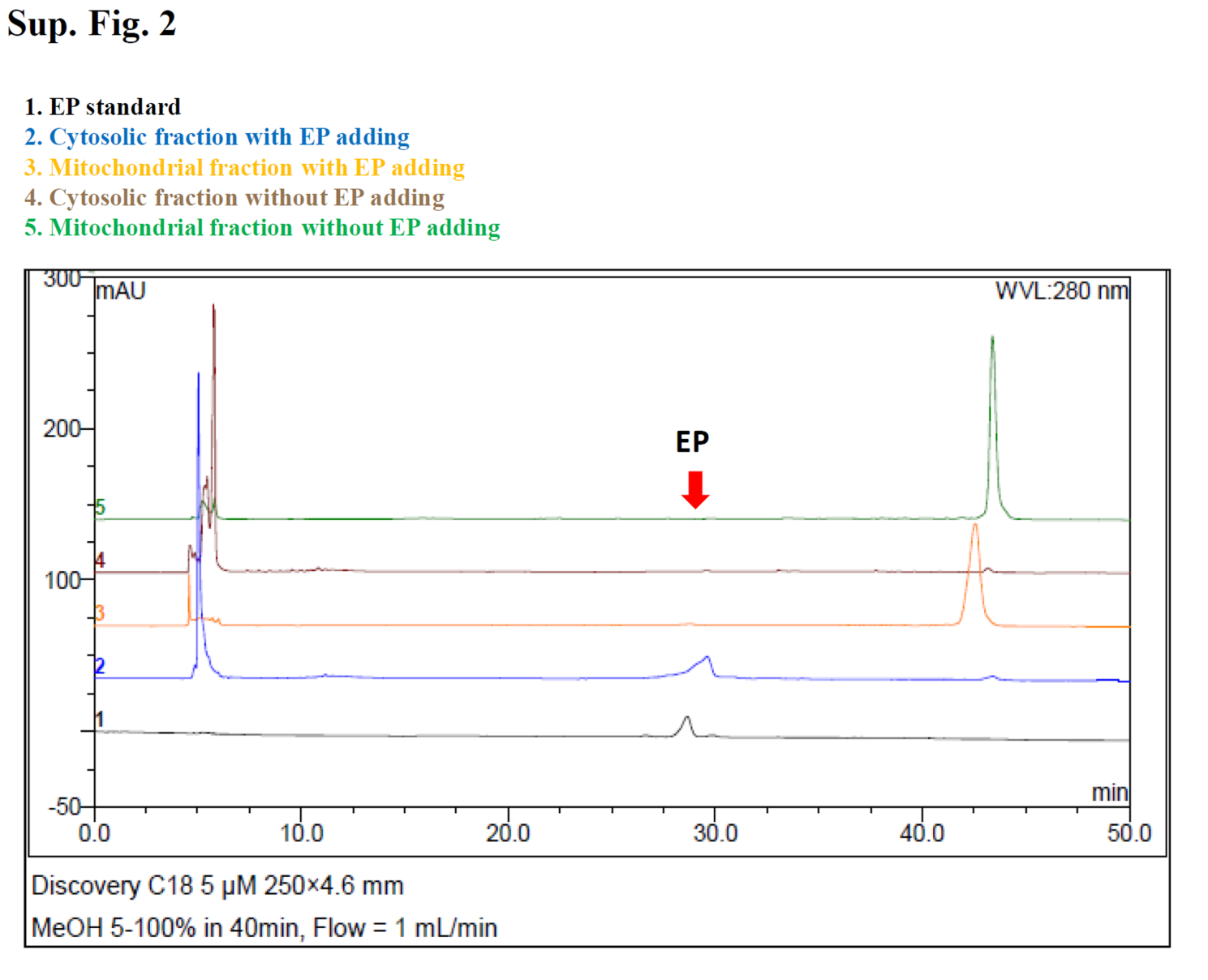


**Figure S2. The entrance of ergosterol peroxide in A549 cells.** A549 cells were treated with 20 μM of EP for 1 h, and then washed with PBS three times. Cells were lysed by RIPA lysis buffer and centrifuge at 14,000 rpm for 20 minutes. The solution was analyzed by HPLC system (Dionex, Thermo Scientific). The black line was EP only as the positive control. Cells were treated with (blue and yellow) or without (purple and green) EP, and also separated into mitochondrial fraction (yellow and green) and cytosolic fraction (blue and purple). The profile indicated the entrance of EP into the cytosol but not into mitochondria.


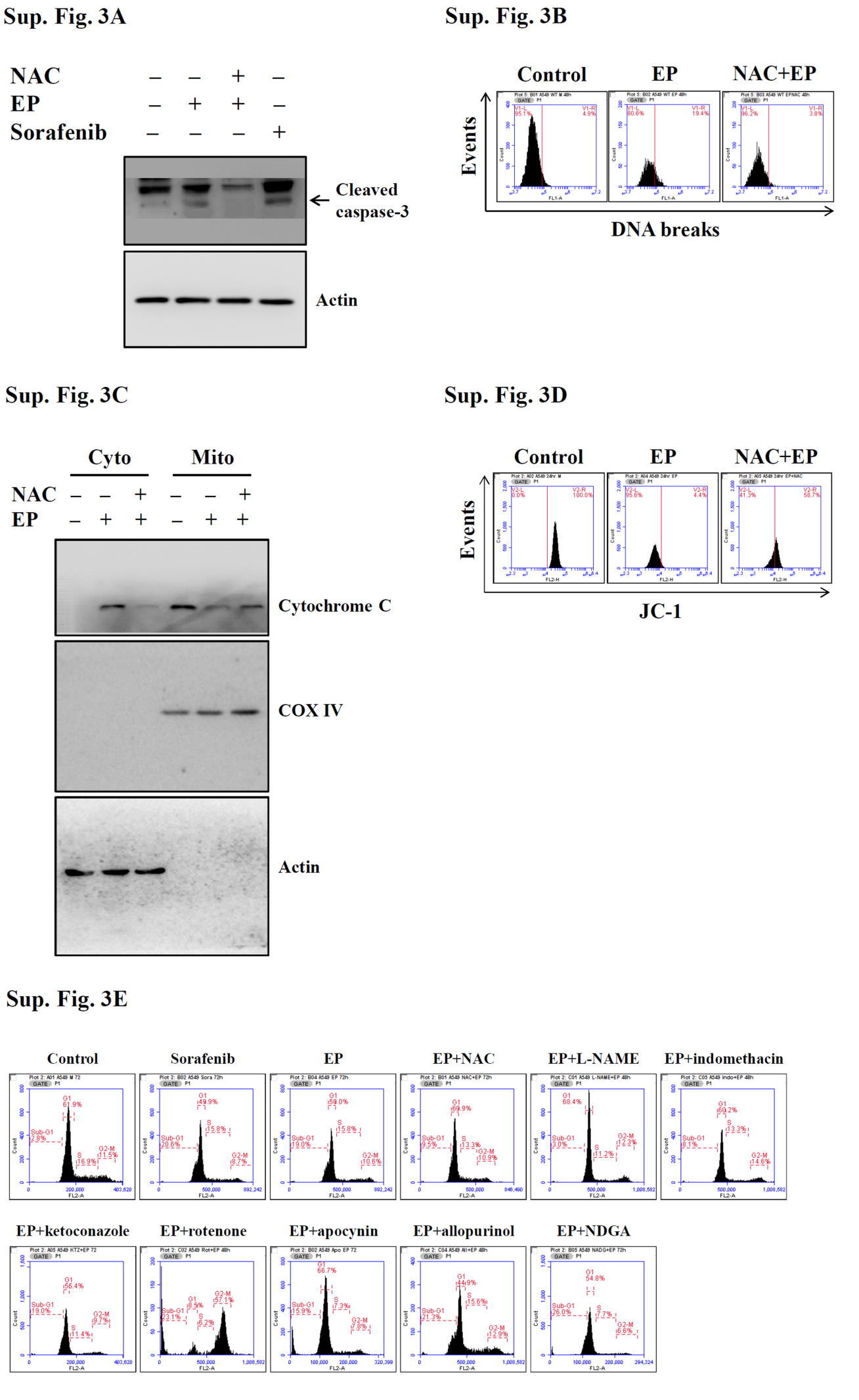


**Figure S3. EP induced ROS-dependent apoptosis.** (A) Effect of NAC on EP-mediated caspase-3 activation. (B) Effect of NAC on EP-mediated DNA breaks. (C) Effect of NAC on EP-mediated cytochrome C release into cytosol. (D) Effect of NAC on EP-mediated mitochondrial membrane potential lost. (E) Effect of ROS generating enzyme inhibitors on EP-mediated sub-G1 phase increase. NAC (10 mM), Ketoconazole (50 μM), NADG (1 μM), apocynin (0.3 μM), rotenone (1 μM), L-NAME (500 μM), Allopurinol (100 μM), Indomethacin (50 μM).


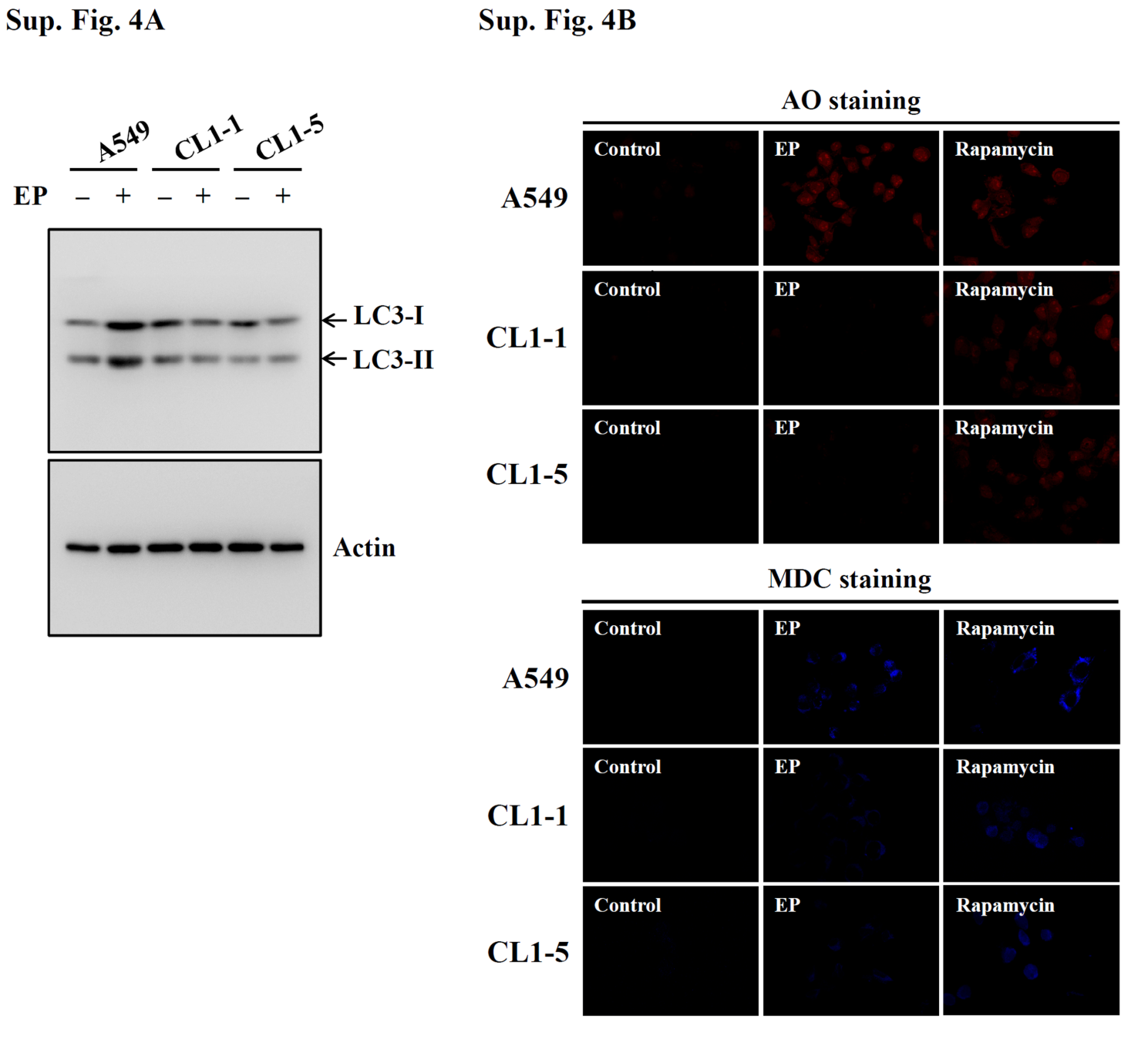


**Figure S4. Effect of EP on autophagy induction in CL1-1 and CL1-5 cells.** (A) Effect of EP on LC3 expression. (B) Effect of EP on AO and MDC staining.


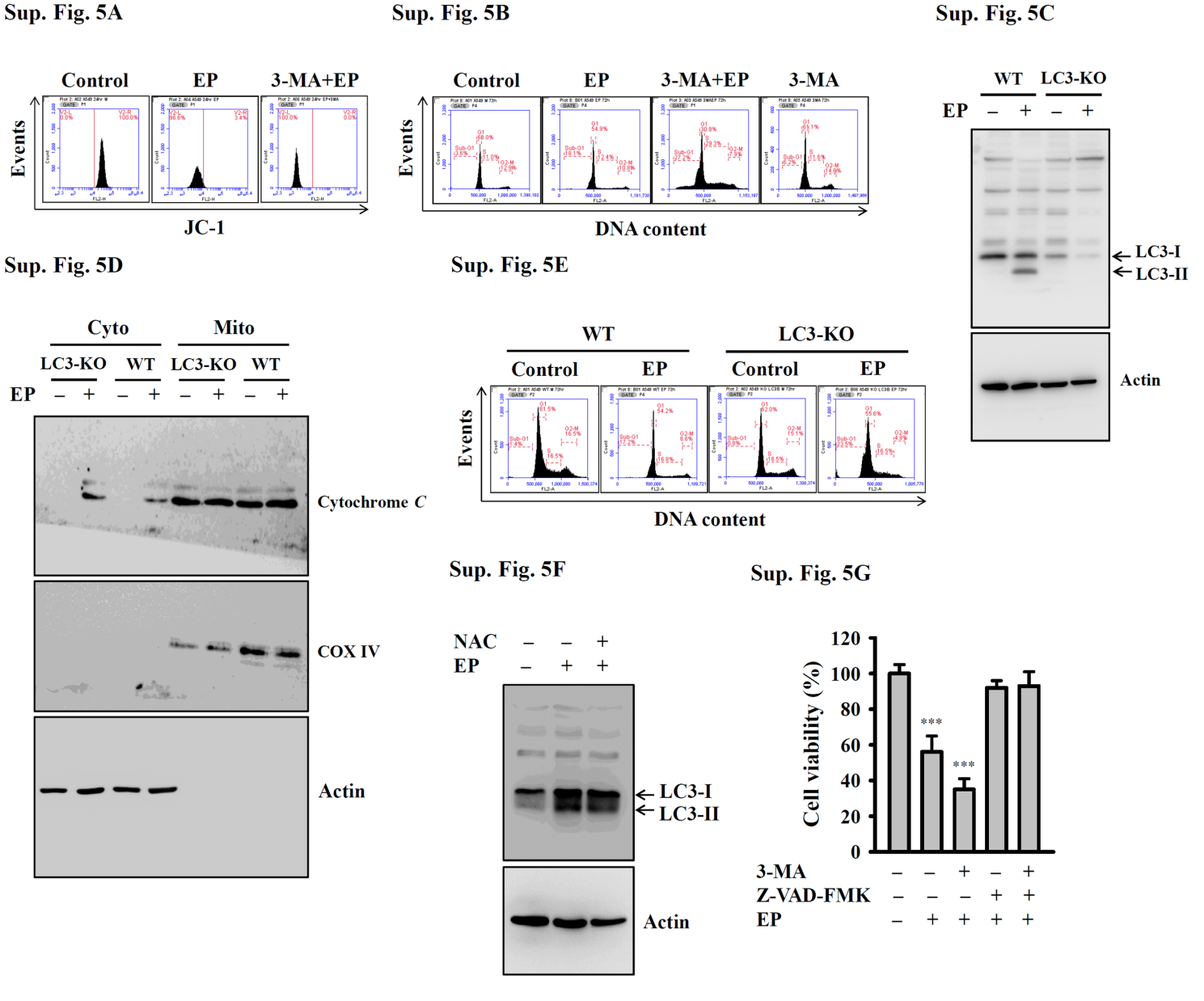


**Figure S5. Effect of autophagy on EP-mediated apoptosis in A549 cells.** (A) Effect of 3-MA on EP-mediated mitochondrial membrane potential (B) Effect of 3-MA on EP-mediated sub-G1 phase (C) Generation of LC3 Knockout A549 cells by CRISPR/Cas9 mediated genome editing. (D) Effect of EP on cytochrome C release into cytosol both in wild-type and LC3 knockout A549 cells. (E) Effect of EP on sub-G1 phase both in wild-type and LC3 knockout A549 cells. (F) Effect of NAC on EP-mediated LC3 expression. (G) Effect of 3-MA and Z-VAD-FMK on the cell viability in EP-treated cells. *** indicate significant differences at the levels of *p* < 0.001 compared to control cells.


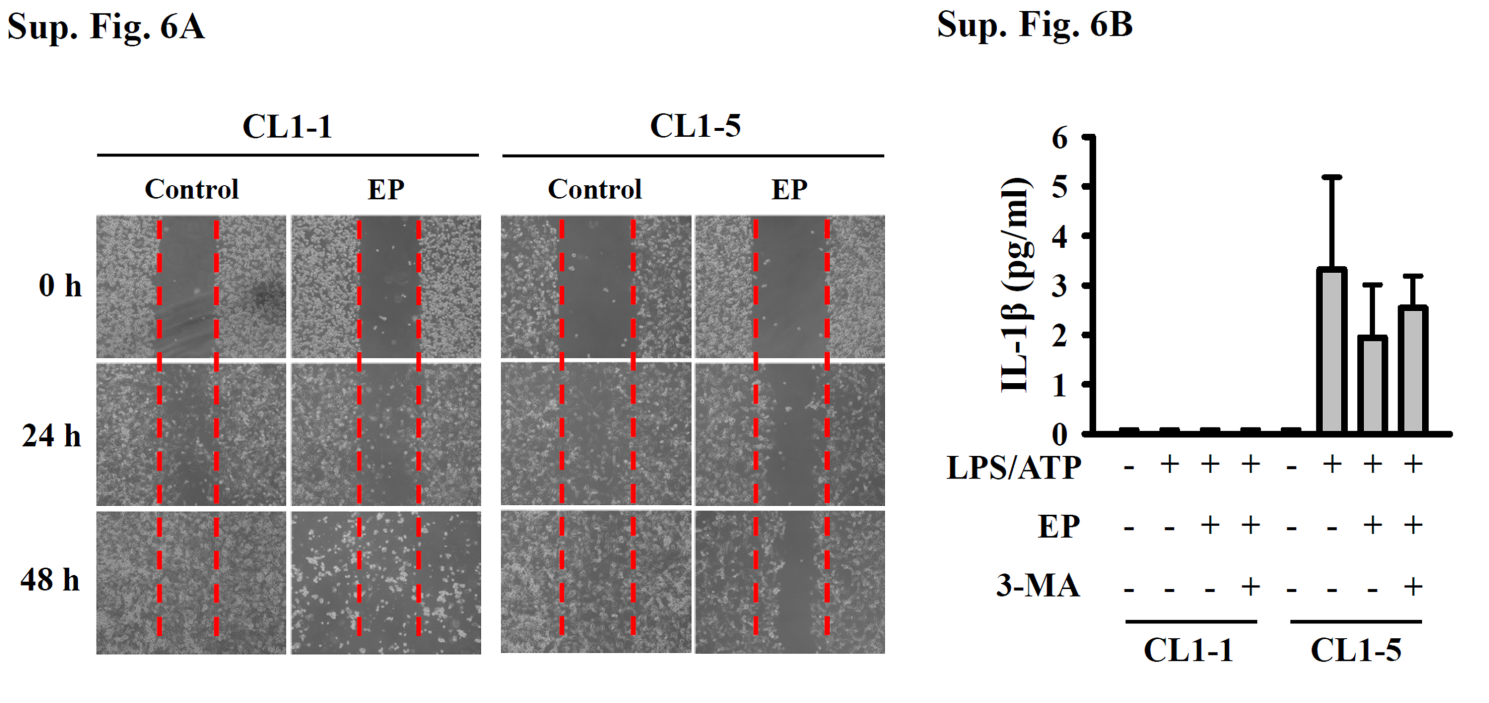


**Figure S6. Effect of EP on the migration and the IL-1β production in CL1-1 and CL1-5 cells.** (A) Effect of EP on migration ability of CL1-1 and CL1-5 cells assayed by scratch assay. (B) Effect of EP on IL-1β production in LPS/ATP-activated CL1-1 and CL1-5 cells.


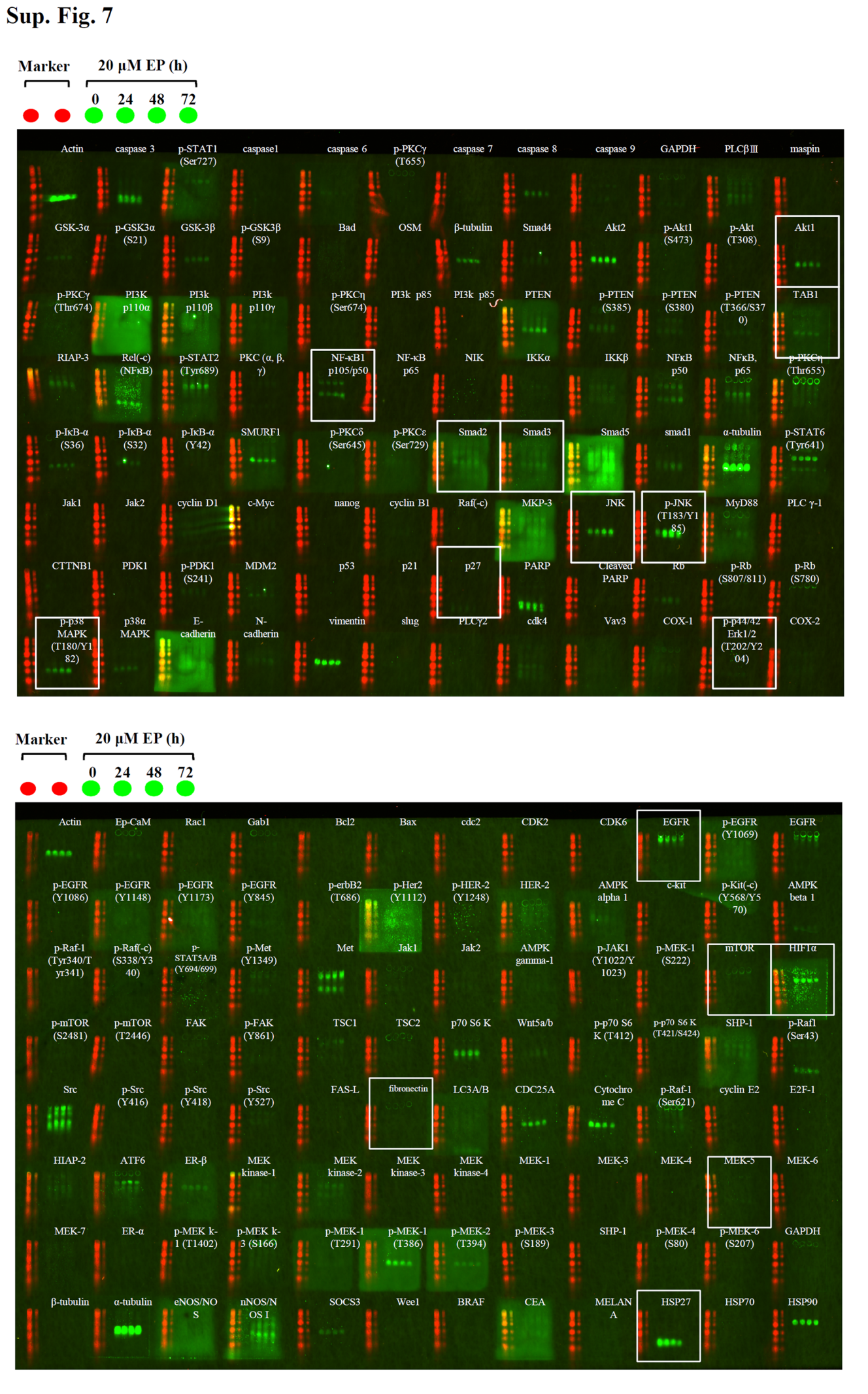


**Figure S7.** Micro-Western Array image and heat-map of abundance fold changes of signaling proteins in A549 cells treated with EP. A549 cells were treated with 20 μM EP for 24, 48, and 72 h. Micro-Western Arrays was performed to measure the changes in abundance and modification of relative cell proliferated signaling proteins. Protein abundance of β-actin was used as loading control. Red color and green color indicated 680 nM and 780 nM wavelength detected by Licor Odyssey scanner for rabbit antibodies and mouse antibodies, respectively. White opened squares indicated the alternations after EP treatment.


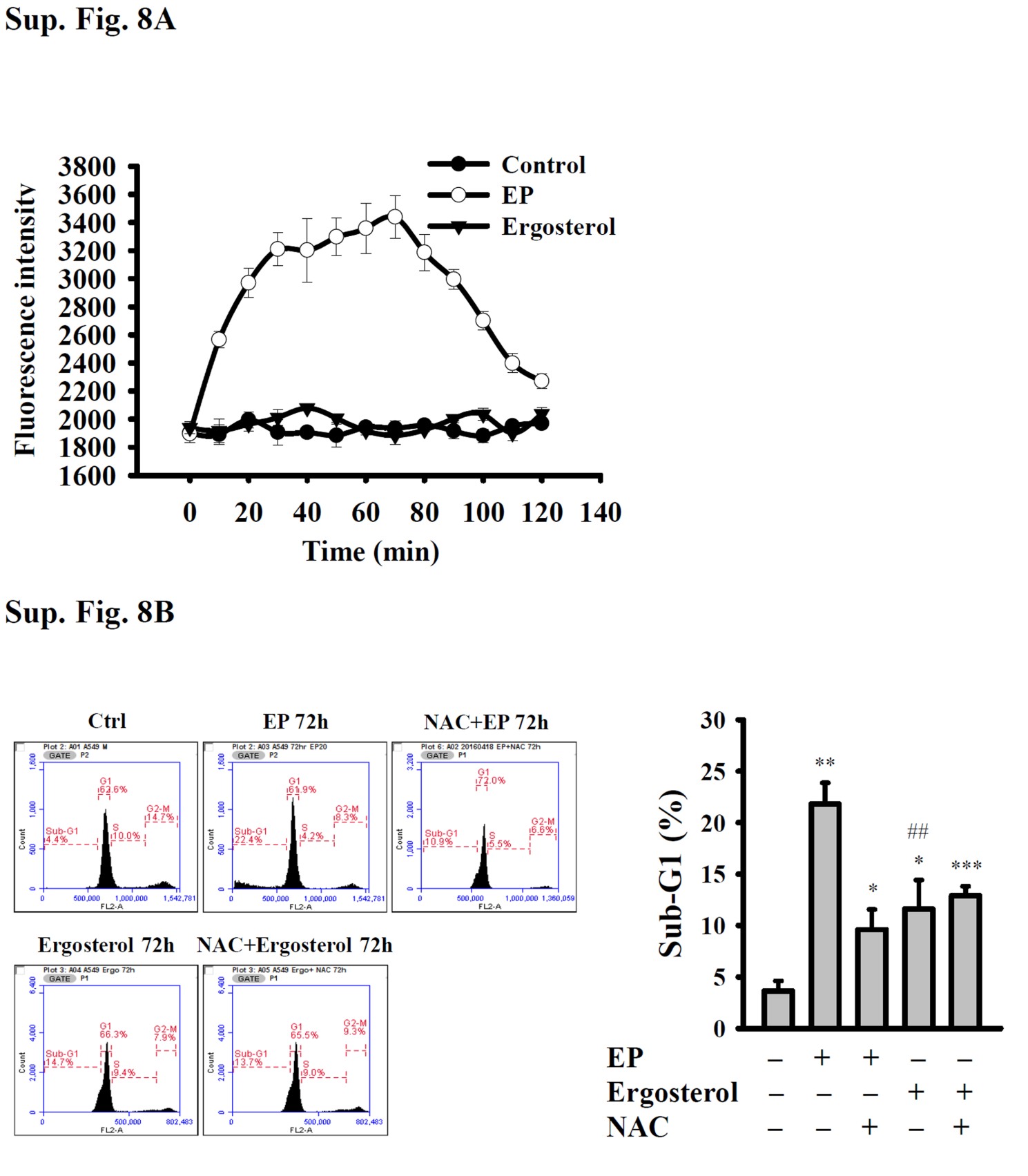


**Figure S8. Effect of Ergosterol and EP on ROS production and apoptosis induction in a549 cells.** (A) Effect of Ergosterol and EP on ROS production. (B) Effect of Ergosterol and EP on cells in sub-G1 phase. *, ** and *** indicate significant differences at the levels of *p* < 0.05, *p* < 0.01 and *p* < 0.001, respectively, compared to control cells. ## indicate significant differences at the levels of *p* < 0.01 compared to EP-treated cells.


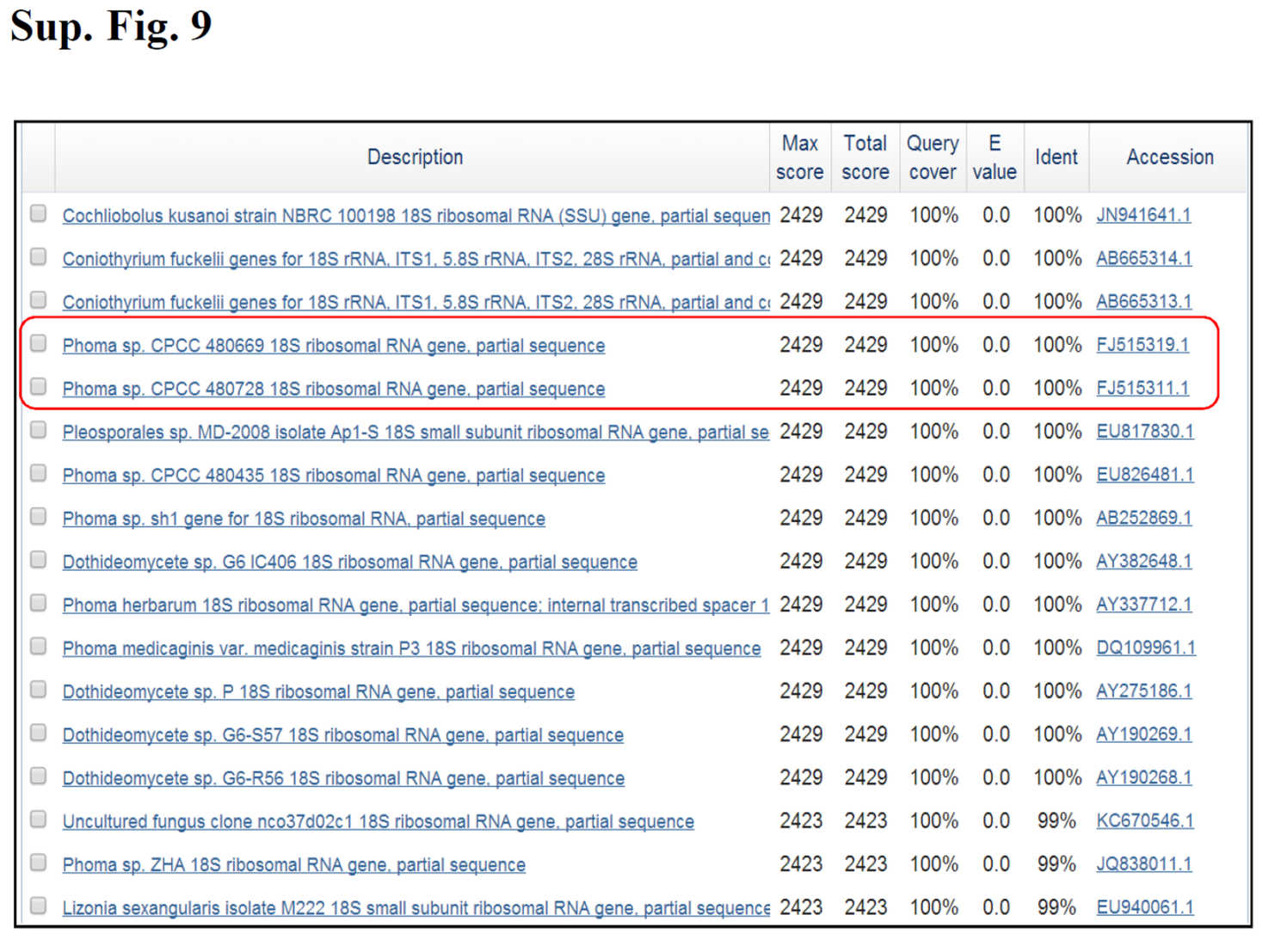


**Figure S9. Classification of *Phoma sp*. was identified by 18S rRNA sequence and by spore morphology.**


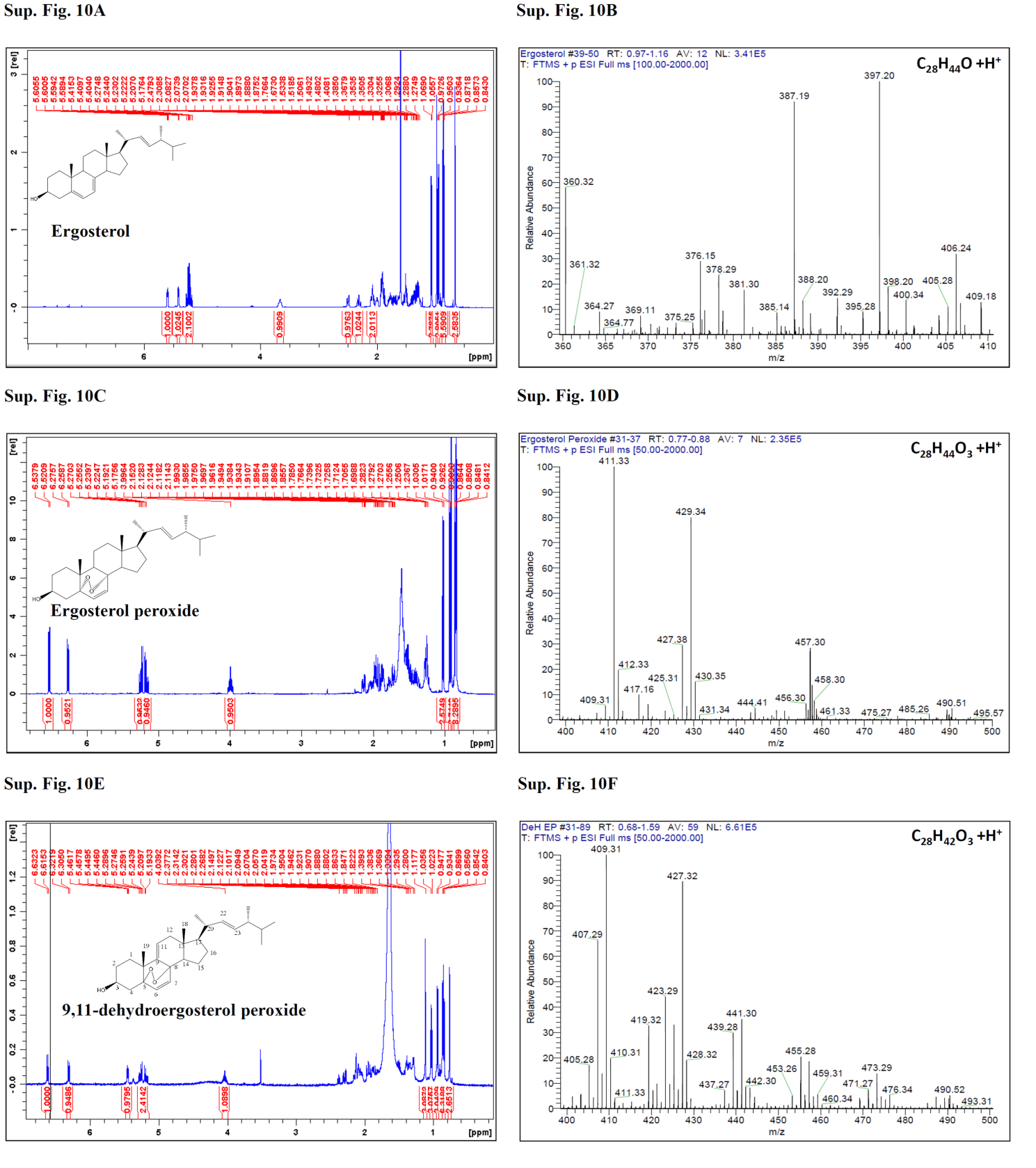


**Figure S10. The identification of Ergosterol, Ergosterol peroxide and 9,11-dehydroergosterol peroxide.** (A) 500MHz 1H-NMR of Ergosterol. (B) LC-ESI-MS of Ergosterol (C_28_H_44_O+H^+^). (C) 500MHz 1H-NMR of Ergosterol peroxide. (D) LC-ESI-MS of Ergosterol peroxide (C_28_H_44_O_3_+H^+^). (E) 500MHz 1H-NMR of 9,11-dehydroergosterol peroxide. (F) LC-ESI-MS of 9,11-dehydroergosterol peroxide (C_28_H_42_O_3_+H^+^).

**Ergosterol**: Structural analysis showed the following parameters: ESI-MS *m/z*: 397.20 [M+H]^+^. 1H NMR (500 MHz, CDCl_3_): δ 0.82 (3H, d, *J* = 6.8 Hz, H-27), 0.83 (3H, s, H-18), 0.84 (3H, d, *J* = 6.8 Hz, H-26), 0.89 (3H, s, H-19), 0.91 (3H, d, *J* = 6.9 Hz, H-28), 1.00 (3H, d, *J* = 6.4 Hz, H-21), 3.97 (1H, tt, *J* = 5.04, 11.47 Hz, H-3), 5.12 (1H, dd, *J* = 8.0, 15.2 Hz, H-22), 5.23 (1H, dd, *J* = 7.6, 15.2 Hz, H-23), 6.24 (1H, d, *J* = 8.4 Hz, H-6), 6.51 (1H, d, *J* = 8.4 Hz, H-7). 13C NMR (100 MHz, CDCl_3_): δ 12.9 (C-18), 17.6 (C-28), 18.2 (C-19), 19.6 (C-21), 19.9 (C-27), 20.6 (C-26), 20.9 (C-11), 23.4 (C-15), 28.6 (C-16), 30.1 (C-2), 33.1 (C-25), 34.7 (C-10), 37.0 (C-1), 37.0 (C-14), 39.3 (C-12), 39.7 (C-20), 42.8 (C-24), 44.6 (C-13), 51.1 (C-4), 51.7 (C-9), 56.2 (C-17), 66.4 (C-3), 79.4 (C-5), 82.2 (C-8), 130.7 (C-24), 132.3 (C-23), 135.2 (C-7), 135.4 (C-22).

**Ergosterol peroxide**: Structural analysis showed the following parameters: ESI-MS *m/z*: 429.34 [M+H]^+^. 1H NMR (500 MHz, CDCl_3_): δ 0.84 (3H, d, *J* = 6.8 Hz, H-27), 0.85 (3H, s, H-18), 0.85 (3H, d, *J* = 6.9 Hz, H-26), 0.90 (3H, s, H-19), 0.93 (3H, d, *J* = 7.2 Hz, H-21), 1.02 (3H, d, *J* = 6.7 Hz, H-28), 1.5 (1H, s, H-9), 1.26 (1H, s, H-11), 1.51 (1H, s, H-11), 3.99 (1H, tt, *J* = 5.02, 11.49 Hz, H-3), 5.16 (1H, dd, *J* = 7.2, 15.6 Hz, H-22), 5.24 (1H, dd, *J* = 7.2, 15.6 Hz, H-23), 6.26 (1H, d, *J* = 8.3 Hz, H-6), 6.52 (1H, d, *J* = 8.3 Hz, H-7). 13C NMR (125 MHz, CDCl_3_): δ 12.9 (C-18), 17.6 (C-28), 18.2 (C-19), 19.6 (C-21), 19.9 (C-27), 20.6 (C-26), 20.9 (C-11), 23.4 (C-15), 28.6 (C-16), 30.1 (C-2), 33.1 (C-25), 34.7 (C-10), 37.0 (C-1), 37.0 (C-14), 39.3 (C-12), 39.7 (C-20), 42.8 (C-24), 44.6 (C-13), 51.1 (C-4), 51.7 (C-9), 56.2 (C-17), 66.4 (C-3), 79.4 (C-5), 82.2 (C-8), 130.7 (C-24), 132.3 (C-23), 135.2 (C-7), 135.4 (C-22).

**9,11-dehydroergosterol peroxide**: Structural analysis showed the following parameters: ESI-MS *m/z*: 427.32 [M+H]^+^. 1H NMR (500 MHz, CDCl_3_): δ 0.84 (3H, d, *J* = 6.8 Hz, H-27), 0.85 (3H, s, H-18), 0.85 (3H, d, *J* = 6.9 Hz, H-26), 0.90 (3H, s, H-19), 0.93 (3H, d, *J* = 7.2 Hz, H-21), 1.02 (3H, d, *J* = 6.7 Hz, H-28), 4.04 (1H, tt, *J* = 5.04, 11.52 Hz, H-3), 5.16 (1H, dd, *J* = 7.8, 17.1 Hz, H-22), 5.24 (1H, dd, *J* = 7.8, 17.1 Hz, H-23), 5.46 (1H, d, *J* = 6.2 Hz, H-11), 6.31 (1H, d, *J* = 8.3 Hz, H-6), 6.63 (1H, d, *J* = 8.3 Hz, H-7).13C NMR (100 MHz, CDCl_3_): δ 12.9 (C-18), 17.6 (C-28), 18.2 (C-19), 19.6 (C-21), 19.9 (C-27), 20.6 (C-26), 20.9 (C-11), 23.4 (C-15), 28.6 (C-16), 30.1 (C-2), 33.1 (C-25), 34.7 (C-10), 37.0 (C-1), 37.0 (C-14), 39.3 (C-12), 39.7 (C-20), 42.8 (C-24), 44.6 (C-13), 51.1 (C-4), 51.7 (C-9), 56.2 (C-17), 66.4 (C-3), 79.4 (C-5), 82.2 (C-8), 130.7 (C-24), 132.3 (C-23), 135.2 (C-7), 135.4 (C-22).


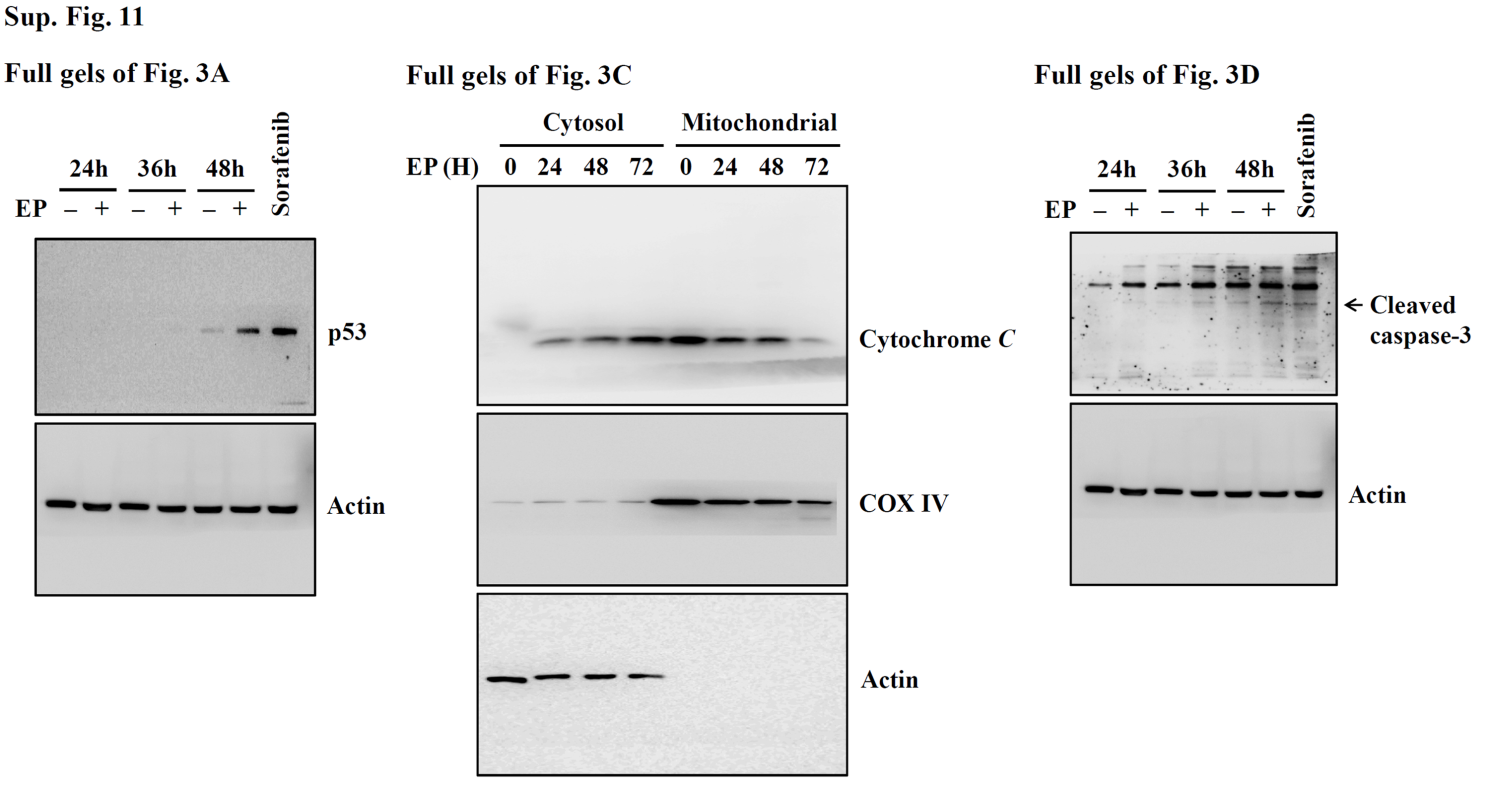


**Figure S11. Full-length blots of Figs. 3A, 3C and 3D.**


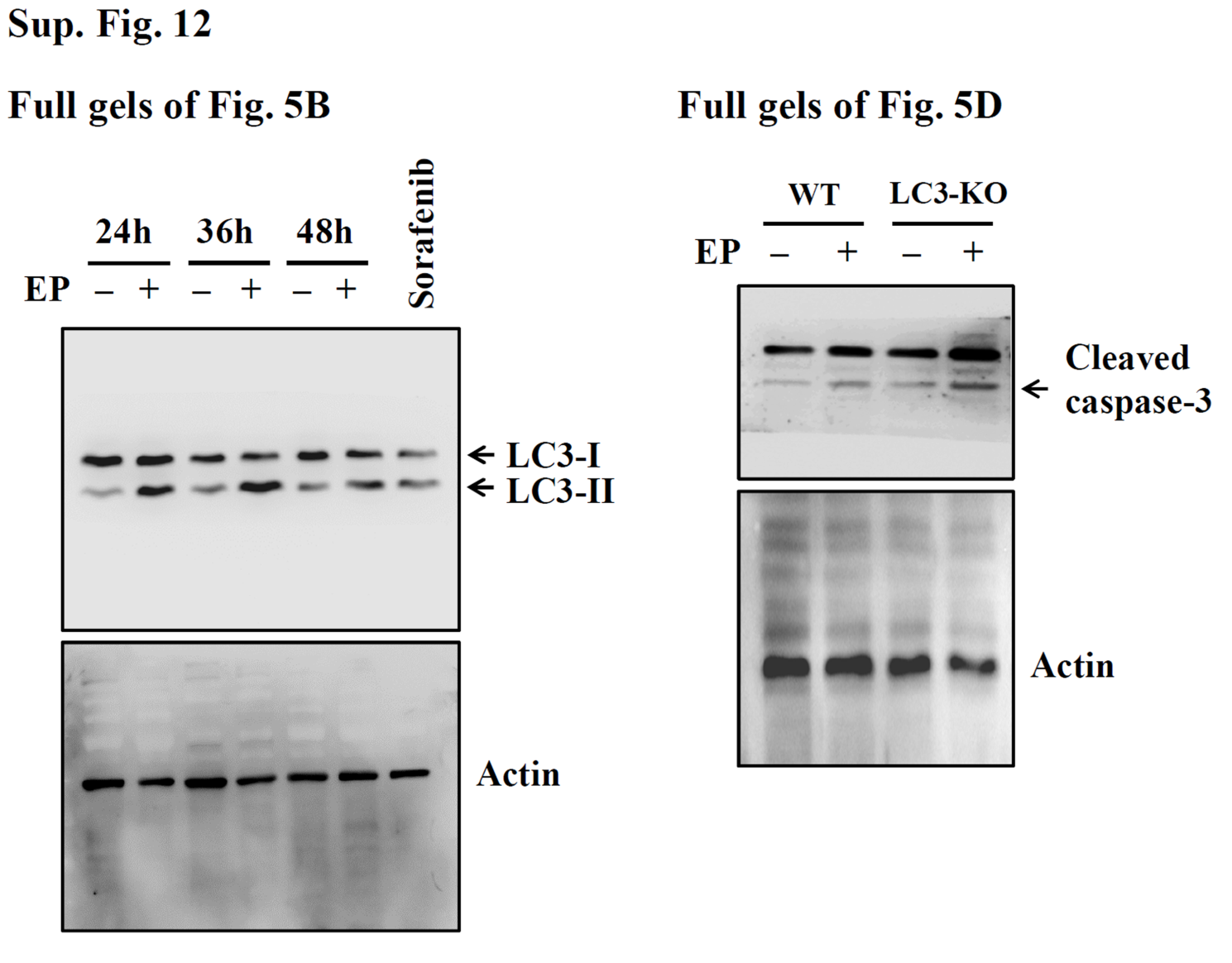


**Figure S12. Full-length blots of Figs. 5B and 5D.**


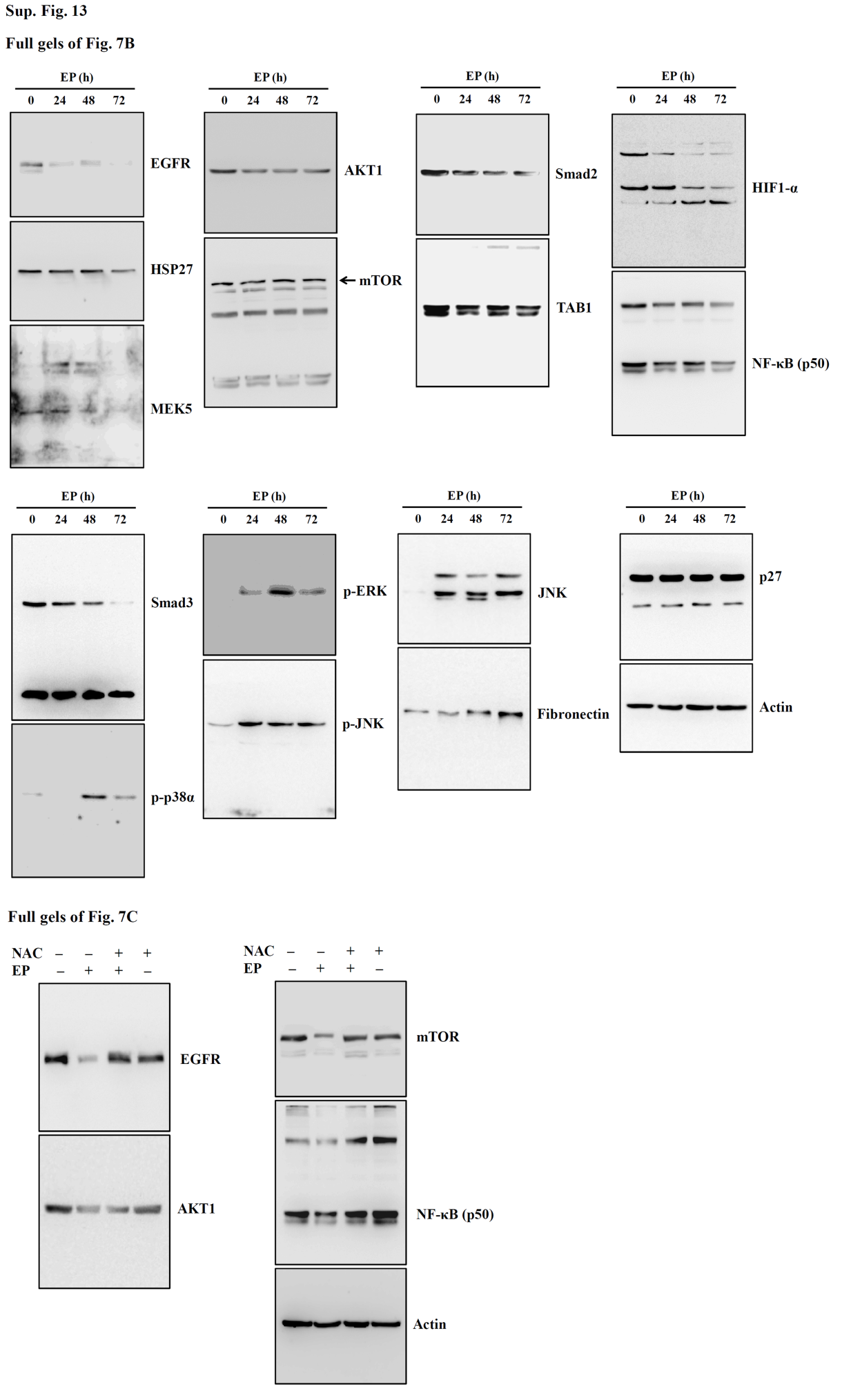


**Figure S13. Full-length blots of Figs. 7B and 7C.**
